# Prevalence of bovine astroviruses and their genotypes in sampled Chinese calves with and without diarrhoea

**DOI:** 10.1099/jgv.0.001640

**Published:** 2021-08-23

**Authors:** Jie Zhu, Mingpu Qi, Chuanwen Jiang, Yongchong Peng, Qingjie Peng, Yingyu Chen, Changmin Hu, Jianguo Chen, Xi Chen, Huanchun Chen, Aizhen Guo

**Affiliations:** ^1^​ State Key Laboratory of Agricultural Microbiology, Huazhong Agricultural University, Wuhan, 430070, PR China; ^2^​ College of Veterinary Medicine, Huazhong Agricultural University, Wuhan, 430070, PR China; ^3^​ Hubei International Scientific and Technological Cooperation Base of Veterinary Epidemiology, Key Laboratory of Preventive Veterinary Medicine in Hubei Province, Wuhan, 430070, PR China; ^4^​ Wuhan Keqian Biology Co.Ltd, Wuhan, 430070, PR China; ^5^​ Key Laboratory of Development of Veterinary Diagnostic Products, Ministry of Agriculture, Huazhong Agricultural University, Wuhan, 430070, PR China; ^6^​ Key Laboratory of Ruminant Bio-products of Ministry of Agriculture and and Rural Affairs, Huazhong Agriculture University, Wuhan 430070, PR China

**Keywords:** bovine astrovirus (BoAstV), cattle, diarrhea, genotypes, genetic evolution, *Mamastrovirus*

## Abstract

Bovine astrovirus (BoAstV) belongs to genus *Mamastravirus* (MAstV). It can be detected in the faeces of both diarrhoeal and healthy calves. However, its prevalence, genetic diversity, and association with cattle diarrhoea are poorly understood. In this study, faecal samples of 87 diarrhoeal and 77 asymptomatic calves from 20 farms in 12 provinces were collected, and BoAstV was detected with reverse transcription-polymerase chain reaction (RT-PCR). The overall prevalence rate of this virus in diarrhoeal and asymptomatic calves was 55.17 % (95 % CI: 44.13, 65.85 %) and 36.36 % (95 % CI: 25.70, 48.12 %), respectively, indicating a correlation between BoAstV infection and calf diarrhoea (OR=2.15, *P*=0.024). BoAstV existed mainly in the form of co-infection (85.53 %) with one to five of nine viruses, and there was a strong positive correlation between BoAstV co-infection and calf diarrhoea (OR=2.83, *P*=0.004). Binary logistic regression analysis confirmed this correlation between BoAstV co-infection and calf diarrhoea (OR=2.41, *P*=0.038). The co-infection of BoAstV and bovine rotavirus (BRV) with or without other viruses accounted for 70.77 % of all the co-infection cases. The diarrhoea risk for the calves co-infected with BoAstV and BRV was 8.14-fold higher than that for the calves co-infected with BoAstV and other viruses (OR=8.14, *P*=0.001). Further, the co-infection of BoAstV/BRV/bovine kobuvirus (BKoV) might increase the risk of calf diarrhoea by 14.82-fold, compared with that of BoAstV and other viruses (OR=14.82, *P* <0.001). Then, nearly complete genomic sequences of nine BoAstV strains were assembled by using next-generation sequencing (NGS) method. Sequence alignment against known astrovirus (AstV) strains at the levels of both amino acids and nucleotides showed a high genetic diversity. Four genotypes were identified, including two known genotypes MAstV-28 (*n*=3) and MAstV-33 (*n*=2) and two novel genotypes designated tentatively as MAstV-34 (*n*=1) and MAstV-35 (*n*=3). In addition, seven out of nine BoAstV strains showed possible inter-genotype recombination and cross-species recombination. Therefore, our results increase the knowledge about the prevalence and the genetic evolution of BoAstV and provide evidence for the association between BoAstV infection and calf diarrhoea.

## Data availability statement

The complete genomes that support the findings of this study are openly available in GenBank accession numbers: MW373712–MW373720.

## Introduction

Astroviruses (AstV) belong to the *Astroviridae* family, including two genera *Mamastravirus* (MAstV) and *Avastrovirus* (AAstV). MAstV can cause diarrhoea and neurological symptoms in mammals, while AAstV can lead to hepatitis, nephritis, and diarrhoea in birds [1]. Among MAstV, human AstV was first discovered to be an etiological agent causing infantile diarrhoea in 1975, while bovine astrovirus (BoAstV) was first reported in 1978 [2]. In 1984, two American bovine strains (US1 and US2) antigenically related to a UK strain (L107) were demonstrated to cause infection and cytopathology of M cells in the dome epithelium [3]. The virulence of BoAstV related to calf diarrhoea was clinically supported in Hong Kong, China, in 2011 [4], the mainland of China in 2013 [5], and Brazil in 2015 [6]. However, some other evidence from calf experiments [2] and natural infection [7] has indicated that BoAstV is avirulent. Up to now, many studies have revealed that BoAstV may cause or exacerbate calf diarrhoea by co-infection with other enteric viruses such as bovine rotavirus (BRV), bovine torovirus (BToV) [3], bovine viral diarrhoea virus (BVDV), bovine coronavirus (BCoV), and bovine kobuvirus (BKoV) [8]. However, the prevalence of BoAstV in diarrhoeal cases varied in different reports, such as 87.5 % in China [5], 85.7 % in Japan [9], 66.7 % in South Korea [8], 64.1 % in Brazil in 2015 [6], and 32.0 % in Egypt [10]. Since 2010, astroviruses have been expanded to at least 25 animal species including various terrestrial domestic animals, wild animals, and aquatic animals. This list includes cattle, pigs, rabbits, dogs, cats, turkeys, chicken, ducks, deer, minks, bats, sea lions, marine fish, etc. [1, 11–13].

At the molecular level, AstVs are a group of small non-enveloped RNA viruses. Their genomes start from a 5′ untranslated region (5′UTR), followed by three open reading frames (ORF1a, ORF1b, and ORF2), a 3′ untranslated region (3′UTR), and a poly (A) tail [11]. According to the latest AstV classification criteria proposed by the International Committee on the Taxonomy of Viruses (ICTV) in 2012, the two genera MAstV and AAstV are further classified into different genotypes based on the mean amino acid genetic distance (*p*-dist) between 0.338 and 0.783 in ORF2 [14]. Specifically, MAstV includes 19 established genotypes (MAstV-1 to 19), 14 proposed genotypes (MAstV-20 to 33), and other undefined genotypes [15].

Although BoAstV is one of the earliest discovered AstV, its genomes remain uncharacterized until four bovine strains from adult cattle without diarrhoea in Hong Kong, China, in 2011 [4] and one strain from yak in the Qinghai–Tibetan Plateau, China, in 2013 [16] were sequenced. So far, a total of 45 genome sequences from seven countries have been publicized in GenBank. Six genotypes (MAstV-13, MAstV-24, MAstV-28, MAstV-29, MAstV-30, and MAstV-33) have been identified from BoAstV isolates mostly based on reverse transcription-polymerase chain reaction (RT-PCR) and sequencing of the ORF2 [17].

However, few studies have addressed the prevalence and genetic diversity of BoAstV and its association with calf diarrhoea, which might be due to the difficulties in isolating this virus from cell and tissue culture and high proportion of co-infection in both diarrhoeal and asymptomatic animals. Fortunately, the current next-generation sequencing (NGS) method can effectively replace the conventional cell culture to identify BoAstV and other co-infected viruses. Considering this, this study is aimed to determine the correlation between BoAstV and calf diarrhoea by investigating the prevalence and genomic characteristics of BoAstVs in Chinese calves with and without diarrhoea based on recent samples collected from a wide range of regions using NGS method. As a result, a strong correlation was observed between BoAstV infection and calf diarrhoea, and two novel genotypes were identified and designated as MAstV-34 and MAstV-35, in addition to the known MAstV-28 and MAstV-33. Our findings would be of significance for developing control measures against BoAstV related to calf diarrhoea.

## Methods

### Sample collection

The 12 provinces were randomly selected from 32 provincial regions in the mainland of China in 2019. Three intensive farms with a scale of over 100 cattle were randomly chosen from each province. First, the occurrence of calf diarrhoea was inquired by phone. In cases of calf diarrhoea occurrence, the farms were visited with the consent of the owners, and faecal samples were randomly collected from both the diarrhoeal and asymptomatic calves based on the principle of case-control study. A total of 164 faecal samples were collected from calves (under 6 weeks old) from 20 farms located in 12 provinces, including 87 from diarrhoeal calves and 77 from asymptomatic calves. The information about the samples was shown in Table 1. All faecal samples were freshly collected from diarrhoeal calves and their asymptomatic controls in the same farms and stored at about 4 °C, and then they were immediately transported to the laboratory where they were stored at −80 °C until use.

**Table 1.** The sampling information on calf faecal collection from farms in China in this study

Regions	Provinces	Farms	Breeds	No. of animal sampled	
				Diarrhoeal calves	Asymptomatic calves
Northeast China	Heilongjiang	A	Dairy	2	5
	Jilin	B	Dairy	3	5
North China	Shandong	C	Dairy	4	3
		D	Dairy	4	5
	Hebei	E	Beef	4	1
		F	Dairy	10	5
		G	Dairy	6	2
	Inner Mongolia	H	Dairy	4	3
		I	Beef	6	5
Central China	Henan	J	Dairy	9	3
		K	Beef	4	2
	Hubei	L	Dairy	3	4
		M	Dairy	3	4
	Hunan	N	Beef	4	2
		O	Beef	4	2
	Jiangxi	P	Beef	4	6
Southwest China	Yunnan	Q	Beef	4	6
Northwest China	Xinjiang	R	Beef	3	4
	Gansu	S	Beef	4	5
		T	Beef	2	5
Total		20		87	77

### BoAstV genome sequencing

One hundred and sixty-four faecal samples from diarrhoea and asymptomatic calves were tested for BoAstV through RT-PCR by previously reported method [7]. BoAstV-positive samples were divided into five regions based on the geographic locations of China (Table 1). Each region contained two sample pools: the asymptomatic faecal sample pool and the diarrhoea faecal sample pool. Each pool contained ten samples with two exceptions (five samples in one pool and 15 samples in the other pool). One gram of faeces was taken from each sample and completely mixed by vortexing. Then, 0.5 g of each sample pool was taken, mixed with 5 ml of sterile phosphate buffer saline (PBS), and fully vortexed. Subsequently, the resultant samples were subjected to two freeze–thaw cycles at −80 °C/25 °C to release virus particles and centrifuged at 8000 ***g*** at 4 °C for 10 min. The supernatant was collected and filtered through 0.22 µm column filters to remove bacteria and other contaminants. According to the manufacturer’s instructions, viral RNA was extracted with a QIAamp Viral RNA/DNA Mini Kit (QIAGEN, Germany), and the rRNA was removed with a QIAseq Fast Select-rRNA HMR Kit (QIAGEN, Germany). Afterwards, the cDNA library was prepared using a QIAseq FX DNA Library Kit (Qiagen, Germany) and commercially sequenced using a MiSeq bench-top sequencer (Illumina) with 151 paired-end reads in Wuhan We Find Biology Co., Ltd (Wuhan, China). The generated raw reads were subjected to quality control by trimming the reads with Phred quality score <10 and by filtering the reads containing adapters. The remaining raw reads were assembled with IDBA [18] and aligned against the Nr (NCBI non-redundant protein sequences) database using an *E*-value ≤0.001. The virome output was visualized and analysed with MEGAN 6 [19].

To validate the genome sequences of nine BoAstV strains obtained from NGS, we performed the extra RT-PCR to amplify the whole genomes containing 5–9 fragments and sequencing the RT-PCR products. Briefly, 5–9 specific primer pairs were designed with the overlapping of 100–400 bp between adjacent fragments based on genome sequences of each virus obtained from NGS (Table S1, available in the online version of this article). Nine calf faecal samples were preliminarily processed, as described above, and then viral RNA was extracted with a QIAamp Viral RNA/DNA Mini Kit (QIAGEN, Germany). Reverse transcription (RT) was performed using the reagents of the PrimeScript II Reverse Transcriptase (Takara, Otsu, Shiga, Japan), following the manufacturer’s protocol. Subsequently, RT-PCR was performed to amplify the genome of each BoAstV strain with specific primers (Table S1). The RT-PCR mixture contained 25 µl of 2×PrimeSTAR Max Premix (Takara, Otsu, Shiga, Japan), 2 µl of cDNA, 19 µl of ddH_2_O, and 2 µl each of the primers (10 mM). The RT-PCR products were directly sequenced in Tsingke Biotechnology Co., Ltd. (Beijing, China). The fragments with weak bands were cloned into the pMD19-T vector and sequenced in Tsingke Biotech. The nearly complete genome sequences of nine BoAstV strains were obtained by assembling the fragment sequences acquired above. Finally, the sequences obtained by NGS were aligned against those obtained by Sanger sequencing to determine the complete genome sequences of nine BoAstV strains. These nine BoAstV genome sequences were submitted to GenBank with accession numbers of MW373712–MW373720.

### Genomic analysis of BoAstV isolates

ORF finder (https://www.ncbi.nlm.nih.gov/orffinder/) was used to search for ORFs of the BoAstV sequences obtained in this study. RNA-dependent RNA-polymerase (RdRp) and the conserved regions of the capsid were localized through NCBI conserved domain search (https://www.ncbi.nlm.nih.gov/Structure/cdd/wrpsb.cgi). The online tool FoldIndex (https://fold.weizmann.ac.il/fldbin/findex) was used to predict the locations of viral proteins associated with the genome (VPg) [20]. The tertiary structure of the RdRp was predicted using I‐TASSER (https://swissmodel.expasy.org/interactive) based on previously published sequences. The tertiary structure obtained from I‐TASSER was visualized with the PyMOL (http://www.pymol.org/).

### Phylogenetic analysis

Multiple sequences were aligned using the Clustal W method with mega 7 [21]. Neighbour-joining analysis of the nucleotide and amino acid sequences in different genomic regions of AstVs strains was performed. The unrooted phylogenetic trees were constructed by mega 7 with bootstrap values calculated for 1000 replicates [22]. The identities between nine BoAstV strains and other AstV strains at levels of both nucleotide and amino acid sequences were examined using the Clustal W algorithm in DNAstar MegAlign software. The information on reference strains, including the strains, species, GenBank accession numbers, isolation time and locations, was shown in Table S2. Mean amino acid genetic distances of ORF2 among known MAstV 1–33, novel MAstV-34, and novel MAstV-35 were calculated using mega 7 with the pairwise deletion option and 1000 bootstrap replicates. Information on the representative strains of each genotype was displayed in Table S3.

Similarity plots were drawn using the SimPlot software package (version 3.5.1) [23]. Bootscan analysis was performed using the neighbour-joining tree model by Kimura two-parameter distance algorithm with a window of 200 bp and a step of 20 bp. To determine possible recombination events, the recombination signals in the whole genome of the BoAstV isolates were measured by the Recombinant Detection Program (RDP4, v4.46) with seven methods (RDP, GENECONV, MaxChi, Bootscan, Chimaera, SiScan, and 3Seq) [24]. Briefly, the complete genome sequences of all BoAstV strains (including the isolates in the current study and representative strains from cattle and other species previously published) were inputted to RDP4 to search for recombination signals. The genomes with significant recombination event (*P* <0.05) derived from at least six methods were presented.

### Co-infected viral detection with RT-PCR

Based on NGS data, the sequences of other viral fragments from vertebrate viruses, plant viruses, and insect viruses were predicted. The possible viruses associated with calf diarrhoea was evaluated by reviewing previous reports [25–28], and RT-PCR was used to verify the existence of nine potential enteric viral pathogens including BVDV [29], BRV [30], BCoV [31], bovine enterovirus (BEV) [32], BToV [33], BKoV [34], bovine norovirus (BNoV) [35], bovine nebovirus (BNeV) [36], and mammalian orthoreovirus (MRV) [37] according to the methods described in the above-mentioned literatures.

### Statistical analysis

A chi-square test was used to assess the correlation between BoAstV infection and calf diarrhoea. The *P* <0.05 were considered statistically significant. Odds ratio (OR) and their 95 % confidence interval (CI) were also used to assess the degree of correlation between BoAstV and calf diarrhoea. Using SPSS software, the binary logistic regression method was used to analyse the effects of BoAstV co-infection with other nine viral pathogens (BVDV, BRV, BCoV, BToV, BNoV, BNeV, BKoV, BEV, and MRV) on calf diarrhoea.

## Results

### Prevalence of BoAstV in diarrhoeal and asymptomatic calves

The faecal samples from 20 farms in 12 provinces were examined with RT-PCR. The results indicated that the overall prevalence rate of BoAstV in all sampled calves was 46.34 % (76/164) (95 % CI: 38.53, 54.28). Specifically, the prevalence rate of BoAstV in the diarrhoeal calves was 55.17 % (48/87) (95 % CI: 44.13, 65.85), while that in asymptomatic calves was 36.36 % (28/77) (95 % CI: 25.70, 48.12), indicating an association between BoAstV infection and calf diarrhoea (*P*=0.024) with OR=2.15 (95 % CI: 1.15, 4.04) (Table 2).

**Table 2.** The association between viruses (BoAstV and BRV) infection and calf diarrhoea

	Diarrhoeal calves	Asymptomatic calves	Total	OR (95 % CI: lower, upper)	*P*-value
BoAstV infection	48	28	76	2.15 (1.15–4.04)	0.024
BoAstV negative	39	49	88		
Total	87	77	164		
BoAstV co-infection	45	20	65	2.83 (1.47–5.56)	0.004
BoAstV negative	39	49	88		
Total	84	69	153		
BRV infection	46	28	74	1.96 (1.05–3.67)	0.041
BRV negative	41	49	90		
Total	87	77	164		
BoAstV +BRV	38	8	46	8.14 (2.44–27.15)	0.001
BoAstV +other virus	7	12	19		
Total	45	20	65		
BoAstV +BRV+BKoV	28	2	30	14.82 (3.05–71.99)	<0.001
BoAstV +other virus	17	18	35		
Total	45	20	65		

Further, the BoAstV existed mainly in the form of co-infection accounting for 85.53 % of the BoAstV infection cases. The prevalence rate of BoAstV co-infection in diarrhoeal and asymptomatic calves was 53.57 % (45/84) (95 % CI: 42.35, 64.53), and 28.99 % (20/69) (95 % CI: 18.69, 41.16), respectively, suggesting a significant difference (*P*=0.004) and an association between BoAstV co-infection and calf diarrhoea (OR=2.83, 95 % CI: 1.44, 5.55) (Table 2). We analysed the data with a binary logistic regression model and found a significant contribution of BoAstV co-infection to calf diarrhoea (OR=2.41, *P*=0.038).

Among the 65 BoAstV co-infection cases, BoAstV co-infection with BRV occupying 70.77 % (46/65) (95 % CI: 45.56, 70.56). Although BRV has been reported as one of the common pathogens causing calf diarrhoea [27, 38], the diarrhoea risk for the calves with BRV infection was only 1.96-fold higher than that for calves without BRV infection (OR=1.96, 95 % CI: 1.05, 3.67; *P*=0.041) (Table 2). However, the diarrhoea risk for the calves co-infected with BoAstV and BRV was 8.14-fold higher than that for calves co-infected with BoAstV and other viruses (OR=8.14, 95 % CI: 2.44, 27.15; *P*=0.001) (Table 2). Therefore, both BoAstV and BRV single infection independently contributed to calf diarrhoea and their co-infection greatly raised the risk of calf diarrhoea.

In diarrhoeal calves, there were 22 co-infection combinations of BoAstV and one to five of nine other viruses with the top three prevalence rates as follows: BoAstV/BRV/BKoV 31.11 % (14/45) (95 % CI: 18.17, 46.65), BoAstV/BRV 13.33 % (6/45) (95 % CI: 5.05, 26.79), and BoAstV/BRV/BEV 6.67 % (3/45) (95 % CI: 1.40, 18.27) (Table 3). In asymptomatic calves, there were only eight co-infection combinations of BoAstV and one to three of five other viruses with top three prevalence rates as follows: BoAstV/BRV 30.00 % (6/20) (95 % CI:11.89, 54.28), BoAstV/BEV 25.00 % (5/20) (95 % CI: 8.66, 49.10), and BoAstV/BRV/BKoV 15.00 % (3/21) (95 % CI: 3.21, 37.89) (Table 3). Further analysis demonstrated that the diarrhoea risk for calves co-infected with BoAstV and BRV/BKoV was 14.82-fold higher than that for calves co-infected with BoAstV and other viruses (OR=14.82, 95 % CI: 3.05–71.99; *P* <0.001) (Table 2).

**Table 3.** Percentage of 22 BoAstV co-infection combination

Diarrhoeal calves			Asymptomatic calves		
Patterns of co-infection	Sample No	Percentages % (95 % CI: up, down)	Patterns of co-infection	Sample No	Percentages % (95 % CI: up, down)
BoAstV +BRV+BKoV	14	31.11 (18.17, 46.65)	BoAstV +BRV	6	30.00 (11.89, 54.28)
BoAstV +BRV	6	13.33 (5.05, 26.79)	BoAstV +BEV	5	25.00 (8.66, 49.10)
BoAstV +BRV+BEV	3	6.67 (1.40, 18.27)	BoAstV +BKoV+BEV	3	15.00 (3.21, 37.89)
BoAstV +BRV+BKoV +MRV	2	4.44 (0.54, 15.15)	BoAstV +BKoV	2	10.00 (1.23, 31.70)
BoAstV +BRV+ BKoV+BEV	2	4.44 (0.54, 15.15)	BoAstV +BRV+BKoV	1	5.00 (0.13, 24.87)
BoAstV +BRV+BEV	2	4.44 (0.54, 15.15)	BoAstV +BRV+BKoV +BEV	1	5.00 (0.13, 24.87)
BoAstV +BRV+BKoV +BEV+MRV	2	4.44 (0.54, 15.15)	BoAstV +BVDV+BKoV	1	5.00 (0.13, 24.87)
BoAstV +BRV+BToV +BNeV+ BKoV+MRV	1	2.22 (0.06, 11.77)	BoAstV +BNoV+BKoV	1	5.00 (0.13, 24.87)
BoAstV +BKoV	1	2.22 (0.06, 11.77)			
BoAstV +MRV	1	2.22 (0.06, 11.77)			
BoAstV +BEV	1	2.22 (0.06, 11.77)			
BoAstV +BVDV+BKoV	1	2.22 (0.06, 11.77)			
BoAstV +BNoV+BKoV	1	2.22 (0.06, 11.77)			
BoAstV +BRV+ BNeV+BKoV	1	2.22 (0.06, 11.77)			
BoAstV +BRV+ BCoV+BKoV	1	2.22 (0.06, 11.77)			
BoAstV +BRV+ BVDV+BKoV	1	2.22 (0.06, 11.77)			
BoAstV +BRV+BCoV+BToV	1	2.22 (0.06, 11.77)			
BoAstV +BRV+BVDV+BCoV+ BToV+BKoV	1	2.22 (0.06, 11.77)			
BoAstV +BRV+BNoV+BNeV+ BKoV+MRV	1	2.22 (0.06, 11.77)			
BoAstV +BRV+BCoV+BToV+BKoV +BEV	1	2.22 (0.06, 11.77)			
BoAstV +BRV+BVDV+BCoV+BKoV +BEV	1	2.22 (0.06, 11.77)			
Total	45			20	

In addition, the overall prevalence rate of BoAstV varied greatly with the individual provinces (each including 1–3 farms), ranging from 14.29 % to 75.00 % in all the sampled calves; while ranging from 25.00–100.00 % in the diarrhoeal calves, and from 0.00–75.00 % in asymptomatic calves (Table S4).

### Genomic characterization and evolution analysis of BoAstV isolates

#### General information

**Fig. 1.**
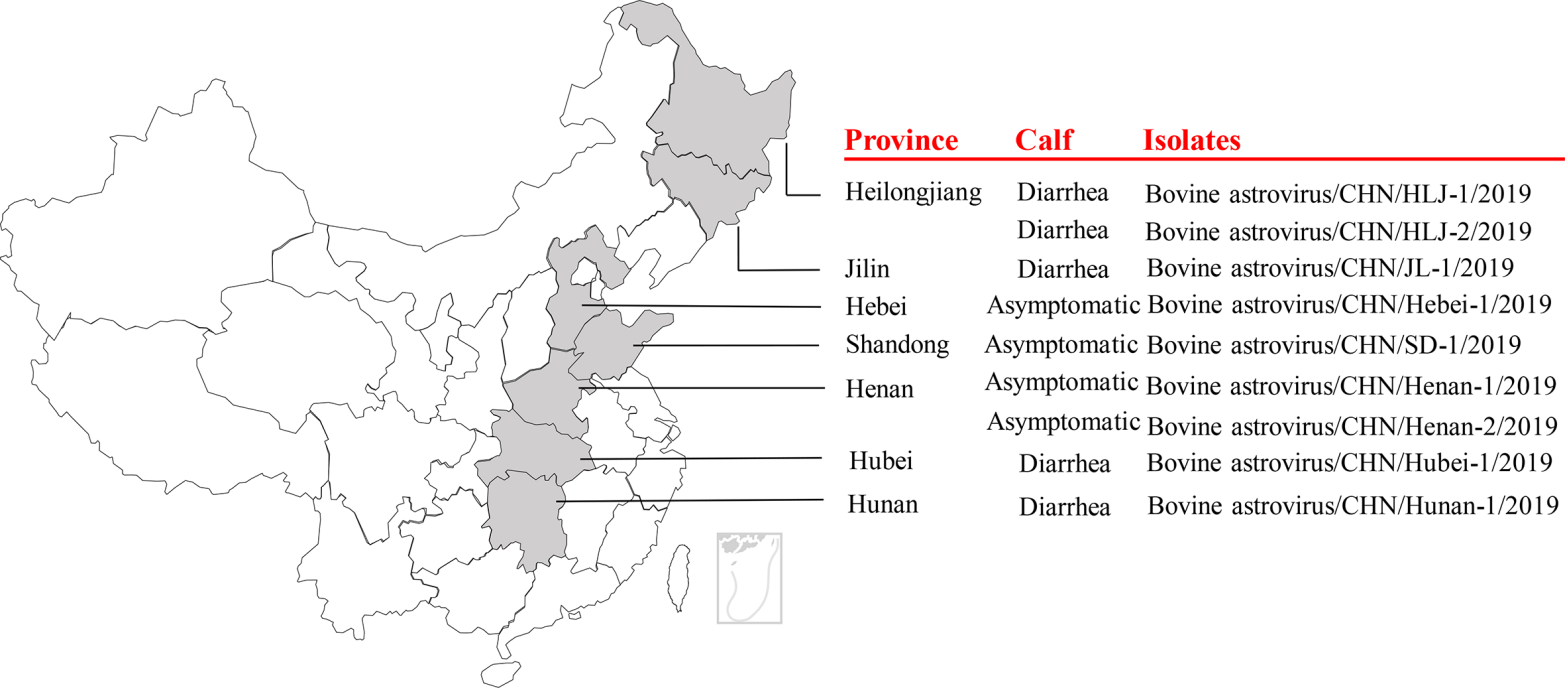
The geographical distribution of nine BoAstV strains. The provinces, calf health status (diarrhoeal /asymptomatic) and names of the isolates were listed in the map right.

Nearly complete genome sequences of nine BoAstV strains (including five strains from diarrhoeal calves and four strains from asymptomatic calves) from NGS data were assembled, and these nine BoAstV strains were distributed in seven provinces in China (Fig. 1). The genome length of these nine strains ranged from 6048 to 6316 nucleotides (nt) excluding the poly(A) tail, and the guanine cytosine (GC) contents ranged from 48–53 % (Table 4). The typical structures of genomes of all the nine BoAstV strains contained one 5′-UTR, three coding region ORFs (ORF1a, ORF 1b, and ORF2), and one 3′-UTR. Like other known astroviruses, these nine BoAstV strains possessed the overlapped region between ORF 1a and ORF 1b with a highly conserved frame-shift ribosomal heptameric sequence (5′-AAAAAAC-3′). In addition, strains BoAstV Henan-1 and Henan-2 exhibited two more amino acids in the highly conserved ORF1b region than seven other BoAstV strains (Table 4).

**Table 4.** Genomic information of BoAstV isolates obtained from deep sequencing in this study

Strains	Abbreviation	Calf clinic signs	GenBank accession no.	Genotypes	Genome length (no polyAtail) (nt)	G+C%	5-′UTR (nt)	ORF 1a		ORF 1b		ORF 2		3-′UTR (nt)
								nt	aa	nt	aa	nt	aa	
Bovine astrovirus/CHN/Henan-2/2019	BoAstV Henan-2	Asymptomatic	MW373718	MAstV-28	6147	53	41	2375	778	1518	505	2234	743	77
Bovine astrovirus/CHN/HLJ-1/2019	BoAstV HLJ-1	Diarrhoea	MW373715	MAstV-28	6262	52	32	2453	817	1512	503	2313	770	56
Bovine astrovirus/CHN/Hebei-1/2019	BoAstV Hebei-1	Asymptomatic	MW373712	MAstV-28	6316	51	34	2454	817	1512	503	2343	780	78
Bovine astrovirus/CHN/HLJ-2/2019	BoAstV HLJ-2	Diarrhoea	MW373714	MAstV-33	6267	53	36	2453	817	1512	503	2316	771	54
Bovine astrovirus/CHN/Hunan-1/2019	BoAstV Hunan-1	Diarrhoea	MW373713	MAstV-33	6288	53	32	2453	817	1512	503	2319	772	76
Bovine astrovirus/CHN/Henan-1/2019	BoAstV Henan-1	Asymptomatic	MW373719	MAstV-34	6140	53	41	2334	778	1518	505	2271	755	84
Bovine astrovirus/CHN/JL-1/2019	BoAstV JL-1	Diarrhoea	MW373720	MAstV-35	6048	48	28	2461	819	1512	503	2162	719*	N/S
Bovine astrovirus/CHN/Hubei-1/2019	BoAstV Hubei-1	Diarrhoea	MW373717	MAstV-35	6190	49	32	2450	816	1512	503	2232	743	68
Bovine astrovirus/CHN/SD-1/2019	BoAstV SD-1	Asymptomatic	MW373716	MAstV-35	6193	49	33	2450	816	1509	503	2240	743	62

*ORF2 sequences were incomplete.

To verify the genome sequences obtained by NGS method from the sample pools, we performed the extra RT-PCR to amplify the whole genomes containing 5–9 fragments (Fig. S1) and sequencing the RT-PCR products of each fragment. We further assembled the fragments of each strain to obtain a nearly full-length genome and found that the re-assembled genome sequences were the same with those generated by NGS (Fig. S2), indicating the reliability of NGS results of pooled samples.

#### Genetic diversity of BoAstV isolates

ORFs’ amino acid and nucleotide sequences and genome-wide sequences of nine BoAstV strains were compared with those of 15 BoAstV reference strains retrieved from GenBank (Table 5). The results indicated that these nine strains were highly divergent from 15 BoAstV reference strains in terms of the genome-wide sequences (39.9–91.1 %) and nucleotide sequences of the three ORFs (ORF1a, 41.1–96.1 %; ORF1b, 55.3–96.6 %; and ORF2, 39.7–87.6 %), respectively. Moreover, the sequence identity at amino acid level was lower than that at nucleotide level for ORF1a, ORF1b, and ORF2 (Table 5). Among the three ORFs, the ORF1b exhibited a higher sequence identity than the other two, indicating that ORF1b was conserved in astroviruses. The genetic distances in the amino acids of ORF2 between nine strains and 43 reference MAstV strains ranged from 0.023 to 0.751 (Table 5).

**Table 5.** Identities at amino acid level of the studied strains of BoAstV with the members of the *Mamastrovirus* genus

Strains	Species	Gene type	Accession no.	Sequence identity (%)							Genetic distance of ORF 2
				Genome	ORF 1a		ORF 1b		ORF 2		
				nt	aa	nt	aa	nt	aa	nt	
BoAstV-Neuro-Uy	Bovine	MAstV-13	MK386569	44.5–46.1	25.6–28.2	42.3–43.7	46.7–48.2	55.3–57.2	33.3–51.6	44.8–56.8	0.702–0.751
BoAstV/JPN/Hokkaido12-25/2009	Bovine	MAstV-24	LC047793	39.9–42.4	21.6–23.5	41.1–43.7	55.2–61.9	57.3–63.5	21.7–25.4	39.7–42.6	0.661–0.686
BoAstV B34/HK	Bovine	MAstV-28	HQ916315	57.2–78.3	53.7–94.6	57.4–81.8	68.9–97.6	66.9–90.3	38.8–76.1	49.7–72.2	0.202–0.591
BoAstV/JPN/Ishikawa24-6	Bovine	MAstV-28	LC047787	55.7–91.1	42.6–98.7	52.9–96.1	68.9–98.8	67.0–96.6	38.8–91.6	50.5–82.6	0.060–0.591
BoAstV B76/HK	Bovine	MAstV-29	HQ916316	54.9–67.4	41.4–75.1	52.9–70.4	69.9–87.9	68.0–78.9	34.6–52.4	47.3–61.4	0.415–0.583
Bovine astrovirus B170/HK	Bovine	MAstV-30	HQ916314	55.2–68.5	41.9–75.6	53.2–70.9	70.5–88.2	68.6–79.5	34.9–53.0	47.8–62.0	0.408–0.580
BoAstV B18/HK	Bovine	MAstV-33	HQ916313	54.8–83.9	41.9–89.1	53.1–94.9	68.9–97.8	67.6–96.5	38.7–90.0	49.9–83.4	0.066–0.572
BoAstV B76-2/HK	Bovine	MAstV-33	HQ916317	55.3–84.6	42.3–97.6	52.9–89.3	69.1–98.0	66.8–89.6	39.0–94.9	51.1–87.6	0.023–0.586
BoAstV/JPN/Kagoshima1-7	Bovine	MAstV-33	LC047796	55.2–80.6	41.9–85.6	53.0–76.6	69.5–96.2	67.0–88.1	39.2–90.0	50.3–83.0	0.070–0.572
BoAstV-GX7/CHN	Bovine	MAstV-33	KJ620979	50.0–80.2	42.6–96.0	52.4–87.6	68.3–97.4	67.0–92.5	39.2–90.0	50.4–83.0	0.174–0.560
Yak astrovirus isolate S8	Yak	MAstV-33	KM822593	54.5–72.7	41.8–91.4	52.6–80.3	68.1–96.4	66.8–87.4	21.3–23.2	37.6–41.4	0.279–0.585
BoAstV/JPN/Hokkaido11-55	Bovine	MAstV-34	LC047790	53.1–77.4	43.9–81.9	50.9–73.0	68.4–96.6	67.2–88.8	34.4–80.5	45.8–76.4	0.145–0.636
BoAstV/JPN/Ishikawa9728	Bovine	MAstV-35	LC047788	54.2–80.6	53.5–85.9	53.1–84.0	68.9–97.2	66.4–89.6	35.7–81.0	45.0–70.0	0.251–0.600
BoAstV/JPN/Hokkaido12-7	Bovine	MAstV-35	LC047791	53.6–85.4	43.6–93.5	53.1–85.9	68.7–98.0	67.0–90.1	34.9–95.4	45.0–87.2	0.047–0.623
BoAstV/JPN/Hokkaido12-18	Bovine	MAstV-35	LC047792	53.9–84.6	43.6–96.1	53.8–87.6	68.9–97.6	65.7–90.0	34.9–91.0	45.0–87.2	0.032–0.621

Likewise, the nine strains also showed significant difference in the sequence identity at the levels of both amino acids and nucleotides, and the genetic distance in the amino acids of ORF2 among these nine strains ranged from 0.049 to 0.557 (Table 6).

**Table 6.** Pairwise nucleotide (upper right) and amino acid (lower left in grey shades) sequence identities (%) among nine BoAstV strains in this study

Strains	Hebei-1	Henan-1	Henan-2	HLJ-1	HLJ-2	Hubei-1	Hunan-1	JL-1	SD-1
	Nonstructural polyprotein 1a/ORF 1a								
Hebei-1	100.0	52.3	52.8	79.7	76.6	67.3	77.3	67.5	66.6
Henan-1	42.2	100.0	89.7	52.7	52.7	53.0	52.2	53.0	52.7
Henan-2	42.3	97.7	100.0	53.3	53.7	53.9	53.2	53.2	53.1
HLJ-1	88.5	42.3	42.8	100.0	75.6	68.4	78.8	68.0	68.0
HLJ-2	84.7	42.2	42.8	84.2	100.0	69.3	77.9	68.2	69.0
Hubei-1	73.3	43.1	43.9	74.0	76.5	100.0	68.7	79.7	86.1
Hunan-1	85.7	42.3	43.1	90.2	85.2	74.9	100.0	67.2	68.0
JL-1	73.2	43.5	44.2	73.4	75.3	90.8	73.9	100.0	78.9
SD-1	73.4	43.1	44.2	73.6	75.8	93.1	74.8	90.9	100.0
	Nonstructural polyprotein 1b/ORF1b								
Hebei-1	100.0	67.4	67.7	87.2	89.1	77.4	93.7	78.0	77.5
Henan-1	68.1	100.0	88.9	67.5	67.4	66.4	66.7	67.0	66.8
Henan-2	68.5	97.0	100.0	66.4	67.8	67.2	67.6	67.6	66.8
HLJ-1	96.6	68.3	68.3	100.0	85.4	78.8	87.1	78.2	78.7
HLJ-2	97.6	68.3	68.7	96.2	100.0	78.1	88.9	78.2	77.7
Hubei-1	88.3	68.9	69.3	87.9	88.5	100.0	77.8	90.1	93.9
Hunan-1	97.2	68.3	68.7	95.6	96.6	87.9	100.0	78.8	78.1
JL-1	88.1	69.7	70.1	86.9	88.5	98.4	88.5	100.0	88.5
SD-1	88.1	69.3	69.7	87.3	88.5	98.0	88.8	98.4	100.0
	Capsid polyprotein /ORF2								
Hebei-1	100.0	50.0	58.6	73.7	56.0	50.5	58.0	50.6	50.1
Henan-1	43.9	100.0	57.1	49.9	50.2	44.5	48.3	43.0	44.8
Henan-2	59.8	47.7	100.0	60.9	48.2	46.9	49.1	46.7	47.4
HLJ-1	78.0	43.7	63.9	100.0	57.5	51.1	60.2	51.2	51.1
HLJ-2	46.7	39.1	38.4	48.7	100.0	51.3	76.2	50.5	52.5
Hubei-1	40.2	35.0	36.5	41.5	44.9	100.0	52.3	73.5	82.0
Hunan-1	50.1	39.4	38.5	51.0	75.5	44.9	100.0	51.1	52.2
JL-1	38.1	33.4	35.4	39.0	45.1	79.1	44.4	100.0	74.3
SD-1	39.1	35.3	36.2	40.7	45.0	91.1	45.6	81.0	100.0
	Complete genomes								
Hebei-1	100	55.5	58.7	79.0	71.7	63.3	74.3	63.5	62.9
Henan-1		100.0	77.6	55.6	55.1	53.5	54.6	53.2	53.6
Henan-2			100.0	59.4	55.3	54.5	55.5	54.4	54.1
HLJ-1				100.0	71.1	64.0	73.9	63.9	64.0
HLJ-2					100.0	64.8	79.7	64.5	64.7
Hubei-1						100.0	64.3	79.6	86.3
Hunan-1							100.0	63.9	64.2
JL-1								100.0	79.2
SD-1									100.0

#### Phylogenetic analysis of BoAstV isolates

**Fig. 2.**
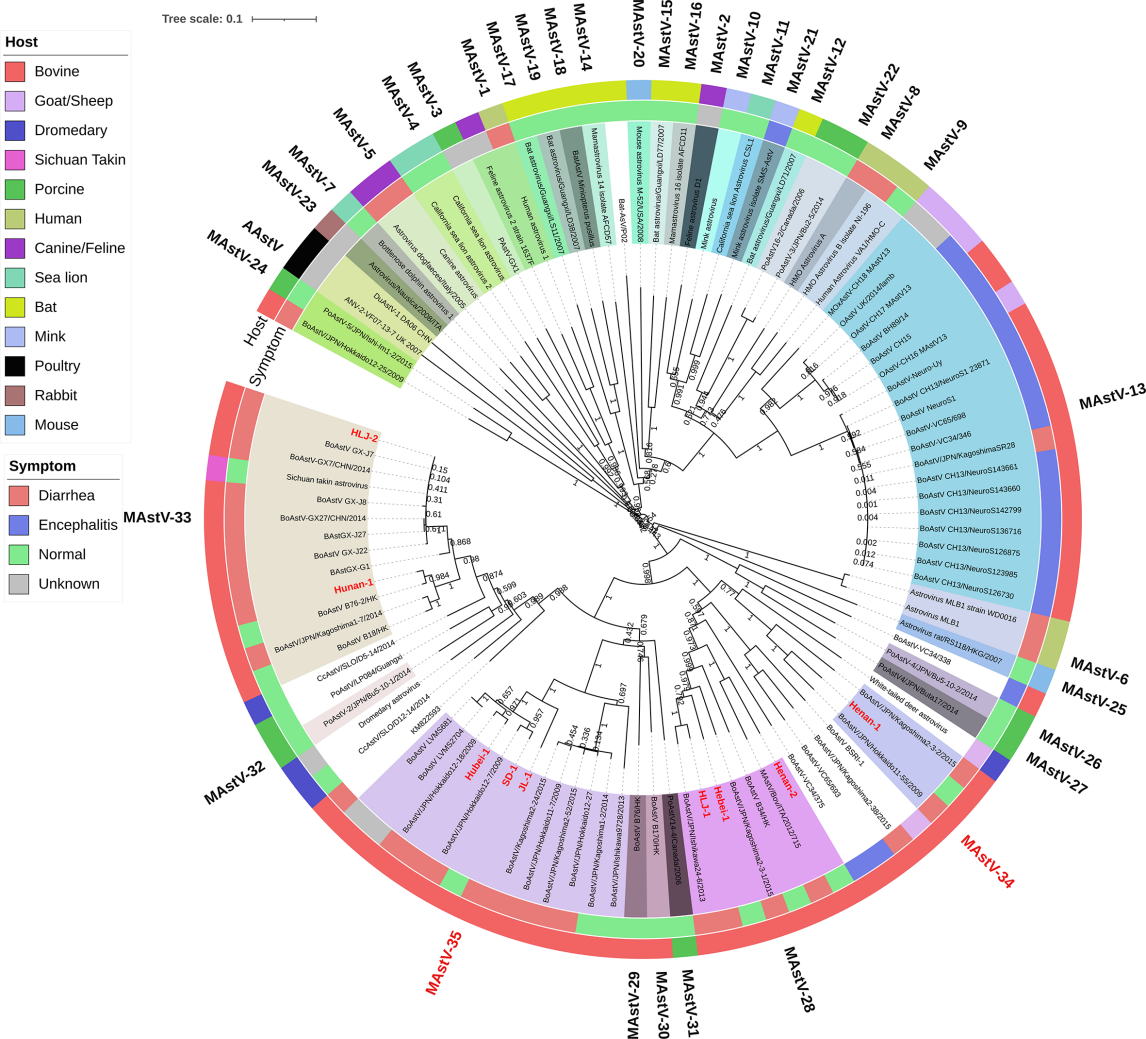
Neighbour-joining phylogenetic tree of AstVs based on ORF 2 amino acid sequences. Unrooted trees were generated using the neighbour-joining method with 1000 bootstrap replicates and sequence alignments were performed by using ClustalW in mega 7.0 software. The strains isolated in this study and the proposed two novel genotypes, MAstV-34 and MAstV-35 were marked in red. Strains were coloured by hosts and indicated by the host and strain names. Information on the reference strains was shown in Table S2.

**Fig. 3.**
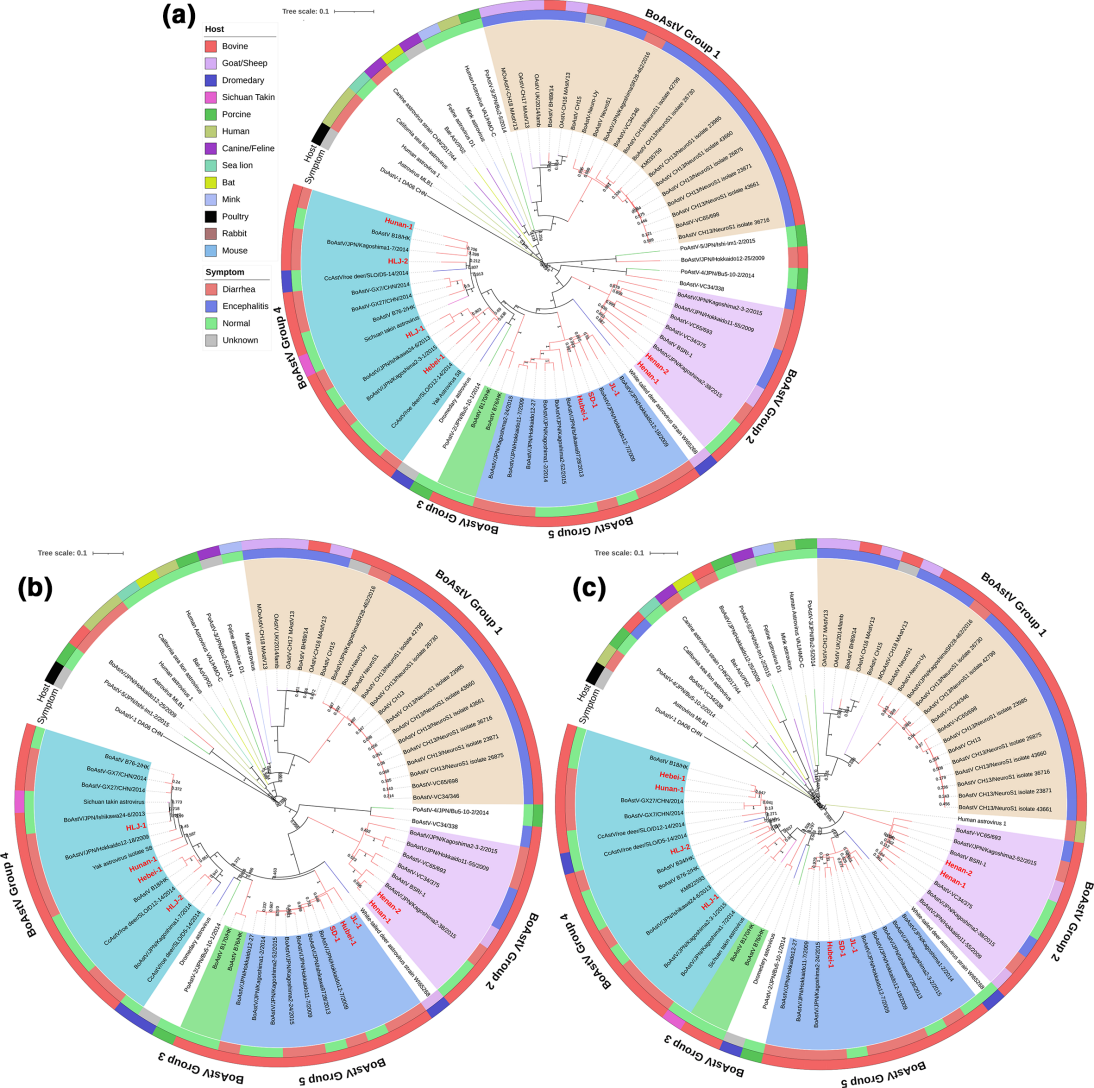
BoAstV strains were separated into five groups based on phylogenetic analysis. Neighbour-joining phylogenetic trees of AstV complete genome nucleotide (**a**), ORF 1a amino acid (**b**) and RdRp amino acid sequences (**c**) were shown respectively. Unrooted trees were generated using the neighbour-joining method with 1000 bootstrap replicates and sequence alignments were performed by using ClustalW in mega 7.0 software. The strains in this study were marked red. BoAstVs were coloured by groups and indicated by the hosts and strain names. Information on the reference strains was shown in Table S2.

To reveal the evolutionary relationship between nine BoAstV strains and 96 other representative AstVs strains retrieved from the GenBank, a phylogenetic tree was constructed based on the nucleotide sequences of complete genomes, and amino acid sequences of RdRp, ORF1a, and ORF2. The results indicated that the nine BoAstV strains fell into four different clusters within the *Mamastrovirus* genus with ORF2 amino acid genetic distance (ranging from 0.338 to 0.783) as the criterion to define different genotypes (Fig. 2) [14]. Strains BoAstV Henan-2, HLJ-1, and Hebei-1 were clustered with Japanese and Italian BoAstV strains, and this cluster was reported as MAstV-28 [1]. Strains BoAstV HLJ-2 and Hunan-1 were clustered with Chinese and Japanese BoAstV strains, and this cluster was previously defined as MAstV-33 [1]. One BoAstV Henan-1 strain and two Japanese BoAstV strains exhibited the closest genetic distance, falling into one cluster, but the genetic distances between these three strains and 33 other known genotype strains ranged from 0.399 to 0.762 (Table 7), thus we identified this cluster as a tentative novel genotype and referred to it as MastV-34. In addition, our data showed that three other strains (BoAstV JL-1, Hubei-1, and SD-1) were clustered together with two BoAstV strains isolated from Uruguay and eight BoAstV strains isolated from Japan. The genetic distance between these strains in this cluster and 33 other known strains ranged from 0.399 to 0.716 (Table 7). Therefore, we identified this cluster as another tentative novel genotype and named it MAstV-35. The ORF2 amino acid genetic distances within these two novel genotypes MAstV-34 and MAstV-35 were 0.128–0.227 and 0.001–0.219, respectively. Furthermore, the genetic distances between the members of MAstV-34 and those of MAstV-35 ranged from 0.404 to 0.507 (Table 7), indicating that our classification of two tentative novel genotypes was highly compliant with ICTV criteria for the determination of novel genotypes. Overall, the nine BoAstV strains in this study were clustered into five groups based on the complete genome nucleotide sequences (Fig. 3a), ORF1a amino acid sequences (Fig. 3b), and RdRp amino acid sequences (Fig. 3c).

**Table 7.** Within genetic distances (*p*-dist) of the ORF2 amino acid sequence for the phylogenetic groups between MAstV 1–33 and novel MAstV 34–35

Genotypes	*p*-dist		
	MAstV 1-33*	MAstV-34	MAstV-35
MAstV 1-33*		0.399–0.762	0.399–0.716
MAstV-34	0.399–0.762		0.404–0.507
MAstV-35	0.399–0.716	0.404–0.507	

The reference strain information for each genotype was shown in Table S3.

#### Recombination analyses

**Fig. 4.**
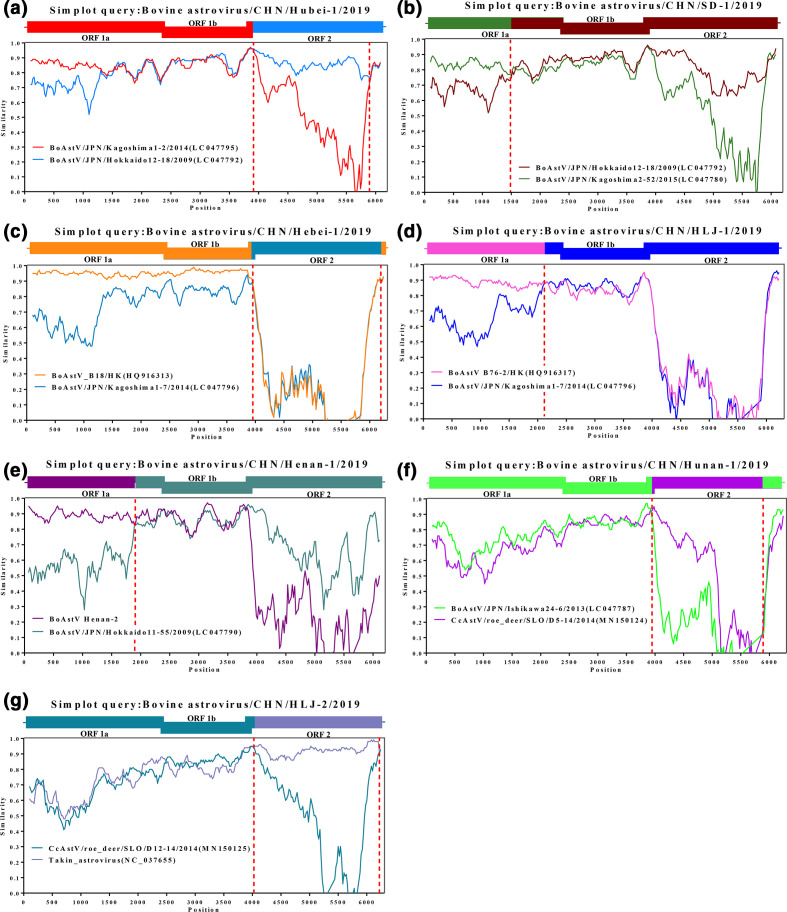
Recombination analysis of the seven BoAstVs genomes. Similarity plots and boot scanning analyses were performed using SimPlot software package (version 3.5.1). Recombination breakpoints were shown with red lines. The recombination events were shown in (a) (BoAstV Hubei-1), (b) (BoAstV SD-1), (c) (BoAstV Hebei-1), (d) (BoAstV HLJ-1), (e) (BoAstV Henan-1), (f) (BoAstV Hunan-1) and (g) (BoAstV HLJ-2), respectively.

The complete genomes of the nine BoAstV strains were aligned against other published AstVs genomes by the ClustalW program in mega 7 for similarity plotting (Fig. 4) and recombination analyses (Table 8). All the nine BoAstV strains were used to identify potential genetic recombination sites with representative strains of other species retrieved from GenBank (until June 2020) as the reference sequences (Table S2). All the other seven BoAstV strains except BoAstV JL-1 and Henan-2 strains were identified to have obvious recombination events by at least six methods in RDP 4 program.

**Table 8.** RDP analysis of recombination events in BoAstV genomes

Astrovirus isolates	Location(nt) (99 % CI]) *	Genetic partner(s) Major Parental/Minor Parental	*P*-value (Seven methods†)							Consistency‡
			RDP	GeneConv	Boot scan	MaxChl	Chimaera	SIScan	3Seq	
BoAstV Hubei-1 (MAstV-35)	4008 (3698–4057), 5826 (5788–6021)	BoAstV/JPN/Kagoshima1-2/2014(MAstV-35), BoAstV/JPN/Hokkaido12-18/2009(MAstV-35)	1.5×10^−40^	6.1×10^−33^	4.4×10^−34^	8.4×10^−28^	2.3×10^−17^	2.3×10^−47^	3.4×10^−15^	**
BoAstV SD-1 (MAstV-35)	16 (5823–28), 1394 (1187–1622)	BoAstV/JPN/Hokkaido12-18/2009(MAstV-35), BoAstV/JPN/Kagoshima2-52/2015(MAstV-35)	1.7×10^−23^	–	2.7×10^−23^	2.2×10^−15^	7.8×10^−20^	4.7×10^−18^	5.7×10^−13^	*
BoAstV Hebei-1 (MAstV-28)	6281 (6246–8), 3925 (3841–3961)	BoAstV/JPN/Kagoshima1-7/2014(MAstV-33), Bovine astrovirus B18/HK(MAstV-33)	2.6×10^−128^	1.3×10^−121^	4.9×10^−126^	4.4×10^−54^	8.9×10^−9^	1.6×10^−67^	1.4×10^−12^	**
BoAstV HLJ-1 (MAstV-28)	8 (6221–23), 1986 (1908–2203)	BoAstV/JPN/Kagoshima1-7/2014(MAstV-33), Bovine astrovirus B76-2/HK(MAstV-33)	2.6×10^−72^	5.6×10^−59^	2.2×10^−68^	5.5×10^−32^	1.2×10^−27^	1.5×10^−40^	2.9×10^−9^	**
BoAstV Henan-1 (MAstV-34)	6117 (6079–31), 1842 (1764–2029)	BoAstV/JPN/Hokkaido11-55/2009(MAstV-34), BoAstV Henna-2(MAstV-28)	1.1×10^−68^	1.0×10^−49^	5.2×10^−69^	1.0×10^−26^	3.9×10^−19^	1.0×10^−46^	9.6×10^−11^	**
BoAstV Hunan-1 (MAstV-33)	3944 (3842–4026), 5928 (4129–6012)	BoAstV/JPN/Ishikawa24-6/2013(MAstV-28), CcAstV/roe_deer/SLO/D5-14/2014(MAstV-33)	1.3×10^−5^	1.3×10^−7^	4.5×10^−10^	3.3×10^−3^	1.2×10^−3^	2.8×10^−8^	2.5×10^−3^	**
BoAstV HLJ-2 (MAstV-33)	4091 (4016–4496), 6254 (6160–59)	CcAstV/roe_deer/SLO/D12-14/2014(Unclassified), Takin astrovirus (MAstV-33)	1.2×10^−53^	4.7×10^−72^	5.9×10^−66^	2.2×10^−36^	6.7×10^−29^	2.9×10^−70^	2.3×10^−5^	**

*Nucleotide localization of the genomic segments implicated in recombination. In some instances, the region is shown in place of a nucleotide position. The 99 % confidence interval (CI) is shown as the nucleotide segment from X to Y.

†The seven tests for recombination were implemented in the RDP4 program.

‡Asterisks indicate the number of methods used to determine statistical evidence of a recombination event: *, six methods; **, seven methods. Genomes in which statistical evidence of a recombination event was obtained with fewer than six methods were not indicated.

The similarity plot and RDP 4 program analysis indicated that both BoAstV Hubei-1 (MAstV-35) and SD-1 (MAstV-35) were identified as recombinants, and that all their parental strains belonged to MAstV-35 (Fig. 4a, b, Table 8), which was a common recombination within the same genotype to produce progeny viruses. In contrast, although both BoAstV Hebei-1 and HLJ-1 strains were identified as MAstV-28 and detected to have significant recombination events, their parental strains belonged to MAstV-33 (Fig. 4c, d, Table 8), suggesting that AstVs parental strains with the same genotype produced the progeny viruses with different genotypes through recombination. In addition, a recombination event was identified in BoAstV Henan-1 strain (MAstV-34), and its parental strains were identified as MAstV-34 and MAstV-28, respectively (Fig. 4e, Table 8). BoAstV Henan-1 strain recombination event supported inter-genotype recombination of AstV. To the best of our knowledge, this is the first report on inter-genotype recombination of BoAstV. In addition, BoAstV Hunan-1 and HLJ-2 strains were identified as MAstV-33, and they were observed to have significant recombination events (Fig. 4f, g, Table 8). The parental strains of BoAstV Hunan-1 belonged to BoAstV (MAstV-28) and roe deer AstV (MAstV-33), and that of BoAstV HLJ-2 belonged to roe deer AstV (unclassified genotype) and Sichuan takin AstV (MAstV-33). The recombination of BoAstV Hunan-1 and HLJ-2 strains illustrated that recombination events in AstV can occur across species and genotypes. The phylogenetic analyses confirmed the recombination events of BoAstV and their parental strains (Fig. S3).

#### Putative protein analyses

**Fig. 5.**
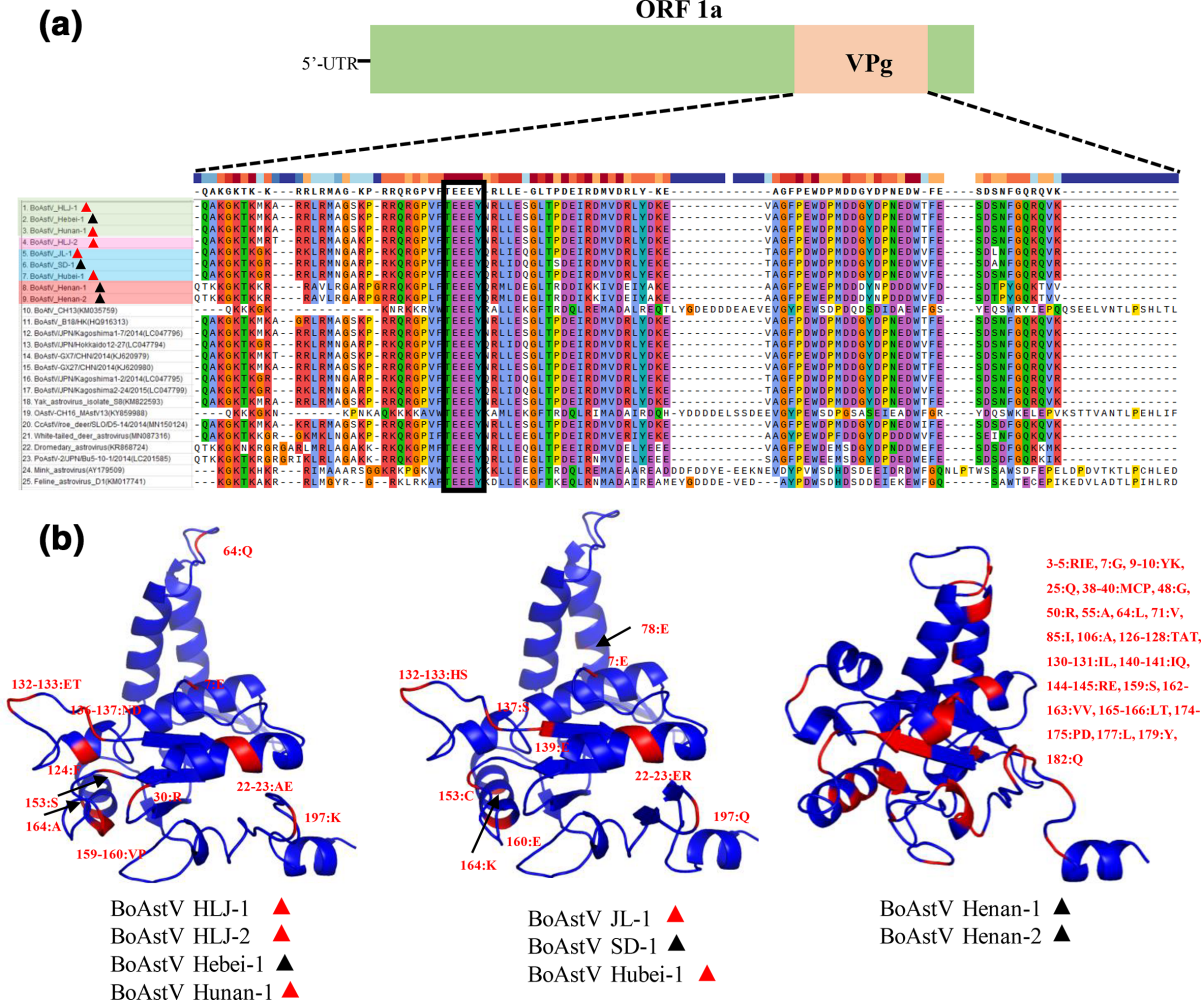
Amino acid (aa) changes in the viral proteins associated with the genome (VPg) and RNA-dependent RNA-polymerase (RdRp) within the nine BoAstV strains. (**a**) Alignment of the partial protein sequences in the VPg regions (end of ORF1a) of MAstV strains. The sequence variation is given with respect to the conserved VPg TEEEY motif (black rectangle). (**b**) Three different cartoon schemes of the RdRp structures. The red and black triangles indicate that the strains were isolated from diarrhoeal and asymptomatic calves respectively.

We further investigated the differences in VPg and RdRp protein sequences (Fig. 5). Although the VPg sequences at the end of ORF 1a were conserved (^689^TEEEY^693^) in MAstV, a few mutations were still observed. Compared with the other seven viruses, BoAstV Henan-1 and Henan-2 had unique mutation sites in VPg, which was similar to mink and dromedary AstV strains. Additionally, the strains were obtained from both diarrhoeal and asymptomatic calves. No correlation was observed between strain mutations and calf health conditions (diarrhoeal vs asymptomatic) (Fig. 5a). The RdRp of BoAstV strains exhibited three types of tertiary structures with two similar to each other and one completely different across the nine strains. No correlation was observed between BoAstV strains’ RdRp tertiary structures and calf health conditions (diarrhoeal vs asymptomatic) (Fig. 5b). In other words, the nine investigated BoAstV strains exhibited genetic diversity in the amino acid sequences of VPg and protein structures of RdRp.

## Discussion

### Co-Infection BoAstV/BRV/BKoV increases the risk of calf diarrhoea

Diarrhoea in animals and humans has been reported to be associated with intestinal AstV infection [11, 39], but whether BoAstV is the causative agent of bovine diarrhoea is controversial [2, 40]. Compared with previous studies of BoAstV, this research focused on the prevalence rate of faecal BoAstV from diarrhoeal and asymptomatic calves with the samples derived from 20 farms in 12 provinces of China based on a case-control study. We reported that BoAstV infection including single infection and co-infection was positively correlated with calf diarrhoea, which was in agreement with previous investigations [6, 16]. Although BoAstV co-infection was previously reported in different countries, such as Korea [8], China [5, 16], and Italy [41], this study first quantified the correlation between different BoAstV co-infection patterns and calf diarrhoea. Our results revealed that the BoAstV/BRV co-infection represented the most frequent co-infection pattern and increased the diarrhoea risk by 8.14-fold; the BoAstV/BRV/BKoV co-infection further increased the diarrhoea risk by 14.82-fold. In addition, BRV has been known as one of the common and important causative agent of calf diarrhoea [27, 38], and its co-infection with BoAstV or even BoAstV/BKoV greatly increased the risk of calf diarrhoea. These findings indicate that the combined vaccine against BoAstV/BRV/BKoV infection might be a potential control measure over calf viral diarrhoea in China.

In addition, the prevalence rate of BoAstV varied greatly with individual provinces. The main factors affecting the prevalence rate in individual provinces lay in farm management measures after the birth of calves such as the proper pen temperature, dry bedding materials, sufficient timely colostrum, and biosafety levels determining the source of the viruses. These farm management measures differed greatly between individual provinces. In addition, provincial difference in prevalence rate might be due to the different sample sizes from one to three farms in each province (Table 1).

### Two novel genotypes of BoAstV are identified

Currently, BoAstVs are composed of five genotypes, namely, MAstV-13 [42–44], MAstV 28–30, and MAstV-33 [4, 5, 7, 9, 41, 45, 46]. In this study, two known genotypes (MAstV-28 and MAstV-33) were determined by directly sequencing the genomes of the faecal samples and two novel genotypes (MAstV-34 and MAstV-35) were identified in terms of the criterion of ORF2 amino acid genetic distance [14]. Consistently, multiple genotypes of AstVs have been identified in other hosts, such as humans, bats, and pigs [1, 47]. However, to date, most BoAstVs have not been assigned to known genotypes (MAstV 1–33) [1, 47]. Therefore, more novel genotypes of BoAstVs might appear in the future. To facilitate the accurate classification of BoAstVs, we attempted to propose a method for classifying BoAstVs in this study based on the phylogenetic analyses of RdRp, ORF1a, and complete genome sequences. This method could cluster most current BoAstV strains (96.08 %, 49/51) into five independent branches, except two strains BoAstV-VC34/338 (accession number: MK987099) and BoAstV/JPN/Hokkaido12-25/2009 (accession number: LC047793). These two strains might be derived from cross-species transmission [9] which was similar to human AstV [48] and porcine AstV [49, 50]. By using our proposed method, seven AstV strains from roe deer, sheep, ovibos moschatus, and Sichuan takin were included and clustered into BoAstV group 1 and group 4. The above-mentioned ruminants were similar to cattle in species or breeding mode, indicating that these strains were closely related to BoAstVs. Since the *Astroviridae* family is an emerging family with significant genetic diversity, long evolution history, and a wide range of host species, the nomenclature and taxonomy of this virus family should be more fully discussed, agreed upon, and updated [47]. Abundant BoAstV gene sequences have been published in GenBank, and most sequences are relatively conservative amplicons with the length of 300–400 bp. Only small fragment sequencing is not conducive to AstVs classification. Therefore, whole genome sequencing is highly suggested when possible. The subsequent comprehensive analysis of multiple regional sequences will be beneficial to classification, like the classification of the rotavirus and influenza virus [51, 52].

### Recombination of strains is identified

Previous studies have shown that recombination events play an important role in the evolution of AstV [53, 54]. In this study, to identify significant recombination signals in the AstV genomes, the recombination events were identified by at least six methods in RDP 4 program coupled with a similarity plotting. In this study, BoAstV Hunan-1 and HLJ-2 strains were both observed to have very obvious recombination signals with their parental strains identified as BoAstV and roe deer AstV (CcAstV), Sichuan takin AstV and CcAstV, respectively. Our results were in line with the previous report on the cross-species recombination between human and California sea lion AstV strains [55], between porcine AstV and human AstV strains [56]. Previously, it was speculated that there was a cross-species recombination event between BoAstV and CcAstV [4]. Our data confirmed that the recombination of BoAstVs occurred between cattle and deer. Recombination has been reported to require co-infection with different viruses in the same host cells [57]. It was worth noting that the roe deer AstV (CcAstV) strain (AstV SLO/D12–14) from Slovenia [58] and the Sichuan takin AstV strain from China [59] were the major and minor parents of the recombinant BoAstV HLJ-2, respectively. Since Sichuan takin and roe deer belonged to completely different species, and they were geographically separately, recombination of these two viruses hinted the occurrence of cross-species cross-geographical regions transmission event, which could be explained as follows. AstVs, as common water pollutants, might spread through the faecal–oral route [60], and water used for breeding cattle could be contaminated by infected wild animals.

Our data showed that four out of seven recombination events occurred in the ORF1b-ORF2 junction region which contained conserved sequences and a stable hairpin structure [61, 62]. In this specific junction region, the recombination of AstVs has been reported in several studies [57, 63–66]. The sequence conservation in this region increased the likelihood of homologous recombination, and the recombination events in the ORF2 region facilitated the swapping of viral capsid protein. Considering the role of capsid protein in defining the cell tropism and host range of the virus, the recombination events in this region might contribute to the separation of virus from its host species [4]. After across-species spreading, the genetic diversity of a virus is greatly increased by adapting to new host species. The genetic diversity of the viruses might further affect their virulence. The findings of virus genetic diversity have great significance for developing a novel vaccine and provide reference for molecular epidemiological studies.

## Conclusion

This case-control study demonstrated that there was an association between BoAstV infection and calf diarrhoea, and a very strong positive correlation between co-infection of BoAstV/BRV/BKoV with calf diarrhoea. Further, the genomes of nine BoAstV strains were sequenced and clustered into four genotypes, including two novel genotypes MAstV-34 and MAstV-35 and two known genotypes MAstV-28 and MAstV-33. Finally, our data confirmed recombination of BoAstVs, which further hinted their cross-species transmission. These findings provide significant epidemiological evidence for developing novel measures to prevent and control BoAstV-induced diarrhoea in calves.
