# Supplementary material for: Prevalence of bovine astroviruses and their genotypes in sampled Chinese calves with and without diarrhoea
Source: J Gen Virol. 2021 Aug 23;102(8):001640. doi: 10.1099/jgv.0.001640 (PMC8513638; doi:10.1099/jgv.0.001640)
Supplement: Supplementary material 1 [file jgv-102-1640-s001.pdf]

Table S1 Primer sequences for verification of complete genomes of nine BoAstV strains

Strains	Fragments	Primers	Sequences (5'-3')	Location in genome (nt)	Size (bp)
Hunan-1	1	Hunan-1-F1<br>Hunan-1-R1	ACCCAGACGGGCGTGTTA<br>AAGTGAGGGAGCGGAGCAT	1645-2274	630
	2	Hunan-1-F2<br>Hunan-1-R2	GCTGATAAGCAGTGGCGTAAA<br>TTCCTCCTGTTCCTCCGTCTA	2753-4069	1317
	3	Hunan-1-F3<br>Hunan-1-R3	GGACAGGCTCTACGACAAGGA<br>GGAAGCAATACCACAAGACACG	2075-2975	901
	4	Hunan-1-F4<br>Hunan-1-R4	GACGGTAAGGTCATATCAAGGGAC<br>GCAGTGGGCAGTTATTGGGTAG	1149-2324	1176
	5	Hunan-1-F5<br>Hunan-1-R5	AGCCGACCGCGTCTTAAC<br>GACCGCAACTGAGCAAACC	203-1240	1038
	6	Hunan-1-F6<br>Hunan-1-R6	GATTAATTAGTTGAGTTCA<br>ATGCATTAGGGCAGCAGCGA	1-350	350
	7	Hunan-1-F7<br>Hunan-1-R7	AGCCGCAAACAGCAGAACA<br>CCGACAACGAAGCCGAAC	3948-5296	1349
	8	Hunan-1-F8<br>Hunan-1-R8	GAGTGGAGACACGATGATGCC<br>TTTTCCCCTTCACCTATGCTAAT	5201-6291	1091
HLJ-2	1	HLJ-2-F1<br>HLJ-2-R1	TAGATAGATCGTGGGTAAGTTGA<br>CCTGCGCCTTGAAACCGA	1-387	387
	2	HLJ-2-F2<br>HLJ-2-R2	GCTCTTGCCTCCGATGACTC<br>AGAAGCCTGTCCCAATGCC	67-1324	1258
	3	HLJ-2-F3<br>HLJ-2-R3	TACACCGTCTTAACAACTACAACAGC<br>ACAGCATGGCAGGGCACA	1102-2359	1288
	4	HLJ-2-F4<br>HLJ-2-R4	GCCTTTGAGTTCGCTTTCCT<br>TCTCCCTTGGTGTTGGTTTTAT	3453-4568	1116
	5	HLJ-2-F5<br>HLJ-2-R5	CGTTGAGAACATGCTCCGTT<br>TCTGGAACACTTGGGGACG	2250-3565	1316
	6	HLJ-2-F6<br>HLJ-2-R6	TACGCATCCACCTACTCCCTATT<br>ACCACTTGCCTTGTTCCACC	4309-5665	1357
	7	HLJ-2-F7<br>HLJ-2-R7	TCATGGCTCATTCGCGGGGGC<br>ACAACGCAAGTGCCATCCT	4909-6230	1322
Hebei-1	1	Hebei-1-F1<br>Hebei-1-R1	AAGGACATTGAAACTCTCT<br>GTGACCAACCAGATTACC	3481-4869	1389
	2	Hebei-1-F2<br>Hebei-1-R2	GACCCAACAACAGGCAAGATAC<br>CTTTCCCCTTCACCTATGCTAAT	4769-6313	1548
	3	Hebei-1-F3<br>Hebei-1-R3	GACGTGGAGAATATGCTCCGTT<br>CACAGTGAACCCGCAGAAGG	2246-3681	1436
	4	Hebei-1-F4<br>Hebei-1-R4	GCTCAACTGCGGTCAACTATG<br>TCAAATGATGGCGGGGAT	1229-2409	1181
	5	Hebei-1-F5<br>Hebei-1-R5	GCCTCCAACGATGAAACCC<br>CCCCTCAATATGGCGAACC	71-1435	1365
Hubei-1	1	Hubei-1-F1<br>Hubei-1-R1	CAAGGCAGGAGGCAACAAG<br>GTGCGTCTGACATAGATGGATAAA	4022-5021	1000
	2	Hubei-1-F2<br>Hubei-1-R2	TAGCGAGCGGGATTATCTTGA<br>GCAGGCGTAGTCGGAACG	2869-4123	1255
	3	Hubei-1-F3<br>Hubei-1-R3	AATGCAACCAGTCAAGTACCCA<br>TTGTTCTGATGCTGCTCAAGG	1806-3092	1287
	4	Hubei-1-F4<br>Hubei-1-R4	GGGGCAAGCAAAATACCAGT<br>CCGAGCACCATTCATCCTAA	1391-1979	589
	5	Hubei-1-F5<br>Hubei-1-R5	CAACTACCACGACTGCCACC<br>ACACCCACTCTGTTTCAATTTTCT	535-1524	990

	6	Hubei-1-F6<br>Hubei-1-R6	TGGGTTCTTGGAGACTGCG<br>AAGCACCCGGTGCTGGG	11-827	769
	7	Hubei-1-F7<br>Hubei-1-R7	CGGGGTCATCCAACTGTCA<br>GCCATCTCCAAGTCACTATCCAC	4769-5890	1122
	8	Hubei-1-F8<br>Hubei-1-R8	GAGTTCCCAACTGGACCTATGC<br>CTGGTAAATCTAATGACCACCGA	5730-6164	435
Henan-2	1	Henan-2-F1<br>Henan-2-R1	AGGGGCGGACCAAAGATG<br>TTGACCAGCGGCAGAACC	3809-5087	1279
	2	Henan-2-F2<br>Henan-2-R2	GGACCTGCTACTTGGACCCC<br>CCCCTCCGTTGTTGTTTCG	2537-4017	1481
	3	Henan-2-F3<br>Henan-2-R3	CCACCCAAGGATGATGAGAAA<br>CACGAAAACCGCCGTAGAA	1638-3050	1413
	4	Henan-2-F4<br>Henan-2-R4	GGAGTGGGTTTCCTATGAGTTGT<br>CTGTGGTGGCATTGGCTGT	203-1032	830
	5	Henan-2-F5<br>Henan-2-R5	GGTTGAGGTCGATACCGAATG<br>ATGGGCTGAGCAGATGGG	4592-5896	1305
	6	Henan-2-F6<br>Henan-2-R6	ACACCTCCAATGATGGGAACTT<br>CACTCGCTGACACCAACTGC	5431-6076	646
	7	Henan-2-F7<br>Henan-2-R7	TGGTGCCGCCTTCTTCAA<br>CTAGGGCCGCGTCCAACT	848-1803	956
	8	Henan-2-F8<br>Henan-2-R8	CTGTCAGAATGTATCTCAACAAG<br>CCCAACAGGCACAGCAAAA	34-445	412
HLJ-1	1	HLJ-1-F1<br>HLJ-1-R1	GCCTTCCTGAACAAGGGGAAG<br>TGCCACTCCGTGTCAACCTC	3461-4693	1233
	2	HLJ-1-F2<br>HLJ-1-R2	GCAAAGGCAGGTCAAGGTC<br>TCTGGAACACTCGGGGAAG	2183-3561	1379
	3	HLJ-1-F3<br>HLJ-1-R3	GGCTTACACCATCATCACAACAC<br>TGCGGCGTACCTATCAACAA	1094-2378	1285
	4	HLJ-1-F4<br>HLJ-1-R4	ACCCGACCGCGTCCTAAC<br>CCTCTTCCTCAATCCCTGCTT	203-1223	1021
	5	HLJ-1-F5<br>HLJ-1-R5	GGCGTCATCAACATCCACTCT<br>CGCAGGTATGCCTCCTATCA	4593-5684	1092
	6	HLJ-1-F6<br>HLJ-1-R6	ACATCACCACTACCCGTATTCAA<br>GCACGCCATCCTAGAGCC	5578-6216	639
	7	HLJ-1-F7<br>HLJ-1-R7	ACCGTGCTGATGAGGCGATAG<br>CAAGGATGGTCGGTTGATGG	49-333	285
Henan-1	1	Henan-1-F1<br>Henan-1-R1	CCATACCCTACTCAGCCAGAAA<br>ACCAACCTCCCTTGAACAGC	3587-4851	1265
	2	Henan-1-F2<br>Henan-1-R2	CCCACAGGCAATCACCAAG<br>CTCCAAAGATACCTCAGGAACTCA	2554-3810	1257
	3	Henan-1-F3<br>Henan-1-R3	GGCTCCATCGTTCAATCTGTC<br>TGGAAATCAGCAAAGGCACA	1449-2652	1204
	4	Henan-1-F4<br>Henan-1-R4	CTGTCAGAATGTATCTCAAC<br>TTCTTTGTCTTCCCCTTCTTAGTTT	34-1840	1807
	5	Henan-1-F5<br>Henan-1-R5	TCGTGGACTTTCAAGGACTACC<br>GCTCGCAAGCAACATCTCATA	4593-5909	1317
	6	Henan-1-F6<br>Henan-1-R6	GGTTATTACTCCTTCAATCGC<br>TCAGCATAGGTGTAGGGGAA	5469-6140	672
JL-1	1	JL-1-F1<br>JL-1-R1	GGTGGACGCCATTCTTCG<br>TGTCTTTTGGTCCTCCCCTC	3125-3928	804
	2	JL-1-F2<br>JL-1-R2	AATGGCACGGGAAATGAAA<br>GAGGACGTAGCGGGTAAGCA	1849-3345	1497
	3	JL-1-F3	GTTT	487-1949	1463

			CTTGGCTTTTCCTTATTTTG		
		JL-1-R3	GCTTCCCCTTTGTTTTCCCT		
	4	JL-1-F4	TTGATGAGTTGCGGTCTATTGC	117-993	877
		JL-1-R4	GAGTGCTGGATGAAGGTGGAA		
	5	JL-1-F5	TTATCAGCCAGTGCCAACCA	3681-4855	1175
		JL-1-R5	AACCATCCATATTATGTCCGAGAC		
	6	JL-1-F6	AAGGGTGATTCAACTGGAGACG	4718-5649	932
		JL-1-R6	TGAACAACTGAGCGATAACAAAA		
	7	JL-1-F7	GGGAATTGGTGAGCTTACAAGATGT	8-299	292
		JL-1-R7	AATTTACTTCCACCGCGGTC		
	8	JL-1-F8	CATTCATTTGCCCATTTTGAG	5402-6019	618
		JL-1-R8	TTGATTGACGGCCATCCTG		
SD-1	1	SD-1-F1	TGCTGGAGATAAGCCCCGTGTA	2936-3886	951
		SD-1-R1	ATTTTAGGCAAGGAGCGACC		
	2	SD-1-F2	GACAAGATTTGATGGCACGATAC	3206-4375	1170
		SD-1-R2	CACTGGAACCAACAAGAGGCT		
	3	SD-1-F3	ACTTTGGGCAACGACAGGTG	2174-3357	1184
		SD-1-R3	TGGGCATGAGGACATAGCG		
	4	SD-1-F4	AAGGTTCCACAGCAAATACAGAGC	1456-2295	840
		SD-1-R4	GCCAACGGCATCAACATCG		
	5	SD-1-F5	GCATCGGGTGCTTTCTATTCT	768-1969	1202
		SD-1-R5	TCATCCTGAGCCTGCGTCTA		
	6	SD-1-F6	CTCTGTCCTCCAATGACCCTG	65-1063	999
		SD-1-R6	GCAAATGTAATGTCCGCAAAA		
	7	SD-1-F7	TCCTTGCCTAAAATTACAGATGAGC	3873-5053	1181
		SD-1-R7	CAGTAGTGGATGTGGCGATGC		
	8	SD-1-F8	GGCTGTTTCGTGGTGGATG	4916-5958	1043
		SD-1-R8	AAGGGACTGTGCTTGAGGCT		
	9	SD-1-F9	GTGGATAGTGATTTGGAGATGGC	5869-6154	286
		SD-1-R9	TGACTACCGAACTGAAACAAAGATG		

Table S2 Information on reference strains retrieved from GenBank and used in the genetic analysis

Strains	Hosts	Symptom	GenBank Accession no.				Time	Locations
			Complete genome	OFR 1a	RdRp	ORF 2		
Bovine astrovirus B18/HK	Bovine	Normal	HQ916313	HQ916313	HQ916313	HQ916313	2011	China
Bovine astrovirus B170/HK	Bovine	Normal	HQ916314	HQ916314	HQ916314	HQ916314	2011	China
Bovine astrovirus B34/HK	Bovine	Normal	HQ916315	HQ916315	HQ916315	HQ916315	2011	China
Bovine astrovirus B76/HK	Bovine	Normal	HQ916316	HQ916316	HQ916316	HQ916316	2011	China
Bovine astrovirus B76-2/HK	Bovine	Normal	HQ916317	HQ916317	HQ916317	HQ916317	2011	China
BoAstV-GX-J27	Bovine	Diarrhea	NA	NA	NA	KJ476832	2013	China
BoAstV-GX-G1	Bovine	Diarrhea	NA	NA	NA	KJ476833	2013	China
BoAstV-GX-J7	Bovine	Diarrhea	NA	NA	NA	KJ476834	2013	China
BoAstV-GX-J22	Bovine	Diarrhea	NA	NA	NA	KJ476835	2013	China
BoAstV-GX-J8	Bovine	Diarrhea	NA	NA	NA	KJ476836	2013	China
BoAstV-GX7/CHN/2014	Bovine	Diarrhea	KJ620979	KJ620979	KJ620979	KJ620979	2014	China
BoAstV-GX27/CHN/2014	Bovine	Diarrhea	KJ620980	KJ620980	KJ620980	KJ620980	2014	China
Takin astrovirus	Sichuan takin	Normal	NC_037655	NC_037655	NC_037655	NC_037655	2013	China
DuAstV-1 _DA06_CHN	duck	Unknown	FJ919225	FJ919225	UF	FJ919225	2016	China
Canine astrovirus strain CHN/2017/44	Canis lupus familiaris	Diarrhea	MF973500	MF973500	UF	MF973500	2017	China
BoAstV/JPN/Hokkaido11-7/2009	Bovine	Diarrhea	LC047789	LC047789	LC047789	LC047789	2009	Japan
BoAstV/JPN/Hokkaido11-55/2009	Bovine	Diarrhea	LC047790	LC047790	LC047790	LC047790	2009	Japan
BoAstV/JPN/Hokkaido12-7/2009	Bovine	Diarrhea	LC047791	LC047791	LC047791	LC047791	2009	Japan
BoAstV/JPN/Hokkaido12-18/2009	Bovine	Diarrhea	LC047792	LC047792	LC047792	LC047792	2009	Japan
BoAstV/JPN/Hokkaido12-25/2009	Bovine	Diarrhea	LC047793	LC047793	LC047793	LC047793	2009	Japan
BoAstV/JPN/Hokkaido12-27	Bovine	Diarrhea	LC047794	LC047794	LC047794	LC047794	2009	Japan
BoAstV/JPN/Ishikawa24-6/2013	Bovine	Normal	LC047787	LC047787	LC047787	LC047787	2013	Japan

BoAstV/JPN/Ishikawa9728/2013	Bovine	Normal	LC047788	LC047788	LC047788	LC047788	2013	Japan
BoAstV/JPN/Kagoshima1-2/2014	Bovine	Normal	LC047795	LC047795	LC047795	LC047795	2014	Japan
BoAstV/JPN/Kagoshima1-7/2014	Bovine	Diarrhea	LC047796	LC047796	LC047796	LC047796	2014	Japan
BoAstV/JPN/Kagoshima2-3-1/2015	Bovine	Diarrhea	LC047797	LC047797	LC047797	LC047797	2015	Japan
BoAstV/JPN/Kagoshima2-3-2/2015	Bovine	Diarrhea	LC047798	LC047798	LC047798	LC047798	2015	Japan
BoAstV/JPN/Kagoshima2-24/2015	Bovine	Diarrhea	LC047799	LC047799	LC047799	LC047799	2015	Japan
BoAstV/JPN/Kagoshima2-52/2015	Bovine	Diarrhea	LC047801	LC047801	LC047801	LC047801	2015	Japan
BoAstV/JPN/Kagoshima2-38/2015	Bovine	Diarrhea	LC047800	LC047800	LC047800	LC047800	2015	Japan
BoAstV/JPN/KagoshimaSR28-462/2016	Bovine	Diarrhea	LC341267	LC341267	LC341267	LC341267	2016	Japan
PoAstV-2/JPN/Bu5-10-1/2014	Porcine	Normal	LC201585	LC201585	UF	LC201585	2014	Japan
PoAstV-3/JPN/Bu2-5/2014	Porcine	Normal	LC201595	LC201595	UF	LC201595	2014	Japan
PoAstV-4/JPN/Bu5-10-2/2014	Porcine	Normal	LC201603	LC201603	UF	LC201603	2014	Japan
PoAstV-5/JPN/Ishi-Im1-2/2015	Porcine	Normal	LC201620	LC201620	UF	LC201620	2014	Japan
Bovine astrovirus NeuroS1	Bovine	Encephalitis	KF233994	KF233994	KF233994	KF233994	2011	USA
Bovine astrovirus isolate BSRI-1	Bovine	Respiratory disease	KP264970	KP264970	KP264970	KP264970	2013	USA
California sea lion astrovirus	sea lion	Normal	JN420358	JN420358	UF	JN420358	2010	USA
Feline astrovirus D1	Feline	Unknown	KM017741	KM017741	UF	KM017741	2013	USA
BoAstV/ITA/2012/715	Bovine	Diarrhea	NA	NA	NA	KT963071	2012	Italy
BoAstV/ITA/2015/954-1	Bovine	Diarrhea	NA	NA	NA	MN718860	2015	Italy
Bovine astrovirus	Bovine	Encephalitis	MN464146	MN464146	MN464146	MN464146	NA	Italy
BoAstV/LVMS681	Bovine	Unknown	NA	NA	NA	MN200262	2015	Uruguay
BoAstV-Neuro-Uy	Bovine	Encephalitis	MK386569	MK386569	MK386569	MK386569	2018	Uruguay
BoAstV/LVMS2704	Bovine	Unknown	NA	NA	NA	MN200263	2016	Uruguay
Bovine astrovirus CH13	Bovine	Encephalitis	KM035759	KM035759	KM035759	KM035759	2012	Switzerland
Bovine astrovirus CH13/NeuroS1 isolate 23871	Bovine	Encephalitis	KX266901	KX266901	KX266901	KX266901	2015	Switzerland

Bovine astrovirus CH13/NeuroS1 isolate 26875	Bovine	Encephalitis	KX266903	KX266903	KX266903	KX266903	2015	Switzerland
Bovine astrovirus CH13/NeuroS1 isolate 36716	Bovine	Encephalitis	KX266904	KX266904	KX266904	KX266904	2015	Switzerland
Bovine astrovirus CH13/NeuroS1 isolate 23985	Bovine	Encephalitis	KX266905	KX266905	KX266905	KX266905	2015	Switzerland
Bovine astrovirus CH13/NeuroS1 isolate 43661	Bovine	Encephalitis	KX266907	KX266907	KX266907	KX266907	2015	Switzerland
Bovine astrovirus CH13/NeuroS1 isolate 43660	Bovine	Encephalitis	KX266908	KX266908	KX266908	KX266908	2015	Switzerland
Bovine astrovirus CH13/NeuroS1 isolate 26730	Bovine	Encephalitis	KX266902	KX266902	KX266902	KX266902	2015	Switzerland
Bovine astrovirus CH13/NeuroS1 isolate 42799	Bovine	Encephalitis	KX266906	KX266906	KX266906	KX266906	2015	Switzerland
Bovine astrovirus isolate CH15	Bovine	Unknown	KT956903	KT956903	KT956903	KT956903	2015	Switzerland
BoAstV-VC34/338	Bovine	Encephalitis	MK987099	MK987099	MK987099	MK987099	2016	Switzerland
BoAstV-VC34/346	Bovine	Encephalitis	MK987100	MK987100	MK987100	MK987100	2016	Switzerland
BoAstV-VC65/698	Bovine	Encephalitis	MK987103	MK987103	MK987103	MK987103	2016	Switzerland
BoAstV-VC34/375	Bovine	Encephalitis	MK987101	MK987101	MK987101	MK987101	2016	Switzerland
BoAstV-VC65/693	Bovine	Encephalitis	MK987102	MK987102	MK987102	MK987102	2016	Switzerland
MOxAstV-CH18 MAstV13	Ovibos moschatus	Unknown	MK211323	MK211323	UF	MK211323	1982	Switzerland
OAstV-CH16 MAstV13	ovine	Encephalitis	KY859988	KY859988	UF	KY859988	2006	Switzerland
CcAstV/roe_deer/SLO/D5-14/2014	roe deer	Normal	MN150124	MN150124	UF	MN150124	2014	Slovenia
CcAstV/roe_deer/SLO/D12-14/2014	roe deer	Normal	MN150125	MN150125	UF	MN150125	2014	Slovenia
OAstV-2/Hungary/2009	Sheep	Normal	NA	NA	NA	JN592482	2009	Hungary
Human astrovirus 1	Human	Diarrhea	HQ398856	HQ398856	UF	HQ398856	2010	Hungary
OAstV/UK/2013/ewe/lib01454	Sheep	Unknown	NA	NA	NA	LT706531	2013	UK
AstV UK/2014/lamb	lamb	Unknown	LT706530	LT706530	UF	LT706530	2014	UK
Human Astrovirus VA1/HMO-C	Human	Normal	KJ920197	KJ920197	UF	KJ920197	2014	UK

ANV-2-VF07-13-7_UK_2007	Avian	Unknown	NA	NA	NA	HQ330482	2007	UK
Bovine astrovirus BH89/14	Bovine	Encephalitis	LN879482	LN879482	LN879482	LN879482	2014	Germany
Astrovirus MLB1	Human	Diarrhea	AB823731	AB823731	UF	AB823731	2010	Bhutan
Dromedary astrovirus	Camelus	Unknown	KR868724	KR868724	UF	KR868724	2013	United Arab Emirates
Bat-AsV/P02	Bat	Normal	MG693176	MG693176	UF	MG693176	2013	Cameroon
Sheep astrovirus	Sheep	Unknown	NA	NA	NA	Y15937	NA	Unknown
Mink astrovirus	Mink	Normal	AY179509	AY179509	UF	AY179509	2002	Unknown

NA: No data; UN:Unreferenced

Table S3 Information on the reference strains retrieved from GenBank and used for the genetic distance of the ORF 2 amino acids

Strains	Genotypes	Accession no.
Human astrovirus 1	MAstV-1	HQ398856
FAstV-D2	MAstV-2	KM017742
PAstV-GX1	MAstV-3	KF787112
California sea lion astrovirus 2	MAstV-4	FJ890352
Astrovirus dogfaeces/Italy/2005	MAstV-5	FM213330
Astrovirus MLB1 strain WD0016	MAstV-6	FJ402983
BdAstV-1_Bottlenose_dolphin	MAstV-7	FJ890355
HMO Astrovirus A	MAstV-8	NC_013443
HMO Astrovirus B	MAstV-9	GQ415661
Mink astrovirus	MAstV-10	AY179509
Mamastrovirus 11	MAstV-11	FJ890351
Bat astrovirus Tm/Guangxi/LD71/2007	MAstV-12	FJ571067
BoAstV-Neuro-Uy	MAstV-13	MK386569
Mamastrovirus 14 isolate AFCD57	MAstV-14	EU847144
Bat astrovirus Tm/Guangxi/LD77/2007	MAstV-15	FJ571066
Mamastrovirus 16 isolate AFCD11	MAstV-16	EU847145
BatAstV_Hipposideros_armiger	MAstV-17	FJ571068
BatAstV_Miniopterus_pusillus_	MAstV-18	EU847155
Bat astrovirus Tm/Guangxi/LD38/2007	MAstV-19	FJ571065
Mouse astrovirus M-52/USA/2008	MAstV-20	JF755422
Mink astrovirus isolate SMS-AstV	MAstV-21	GU985458
PoAstV16-2/Canada/2006	MAstV-22	HM756261
RabAstV_Rabbit	MAstV-23	JN052023
BoAstV/JPN/Hokkaido12-25/2009	MAstV-24	LC047793
Astrovirus rat/RS118/HKG/2007	MAstV-25	HM450381
PoAstV4/JPN/Bu5-10-2/2014	MAstV-26	LC201603
PoAstV4/JPN/Buta17/2014	MAstV-27	LC201604
BoAstV/JPN/Ishikawa24-6	MAstV-28	LC047787
BoAstV B76/HK	MAstV-29	HQ916316
Bovine astrovirus B170/HK	MAstV-30	HQ916314
Astrovirus swine/PoAstV14-4/Canada/2006	MAstV-31	HM756260
Porcupine astrovirus Hb/LP084/Guangxi	MAstV-32	KJ571486
BoAstV B76-2/HK	MAstV-33	HQ916317

Table S4 Test prevalence of AstVs in diarrheal and asymptomatic calves in various regions of China

Regions	Provinces	Test prevalence (95% CI: down, up)		
		Diarrhea calves	Asymptomatic calves	Total
Northeast China	Heilongjiang	100.00% (15.81-100.00)	40.00% (5.27-85.34)	57.14% (18.41-90.10)
	Jilin	100.00% (29.24-100.00)	40.00% (5.27-85.34)	62.50% (24.49-91.48)
North China	Shandong	25.00% (3.19-65.09)	25.00% (3.19-65.09)	25.00% (7.27-52.38)
	Hebei	45.00% (23.06-68.47)	75.00% (34.91-96.81)	53.57% (33.87-72.49)
	Inner Mongolia	70.00% (34.75-93.33)	0.00% (0.00-36.94)	38.89% (17.30-34.25)
Central China	Henan	46.15% (19.22-74.87)	60.00% (14.66-94.73)	50.00% (26.02-73.98)
	Hubei	50.00% (11.81-88.19)	62.50% (24.49-91.48)	57.14% (28.86-82.34)
	Hunan	75.00% (34.91-96.81)	75.00% (19.41-99.37)	75.00% (42.81-94.51)
	Jiangxi	100.00% (39.76-100.00)	33.33% (4.33-77.72)	60.00% (26.24-87.84)
Southwest China	Yunnan	50.00% (6.76-93.24)	50.00% (11.81-88.19)	50.00% (18.71-81.29)
Northwest China	Xinjiang	33.33% (0.84-90.57)	0.00% (0.00-60.24)	14.29% (0.39-57.87)
	Gansu	50.00% (11.81-88.19)	0.00% (0.00-30.85)	18.75% (4.05-45.65)

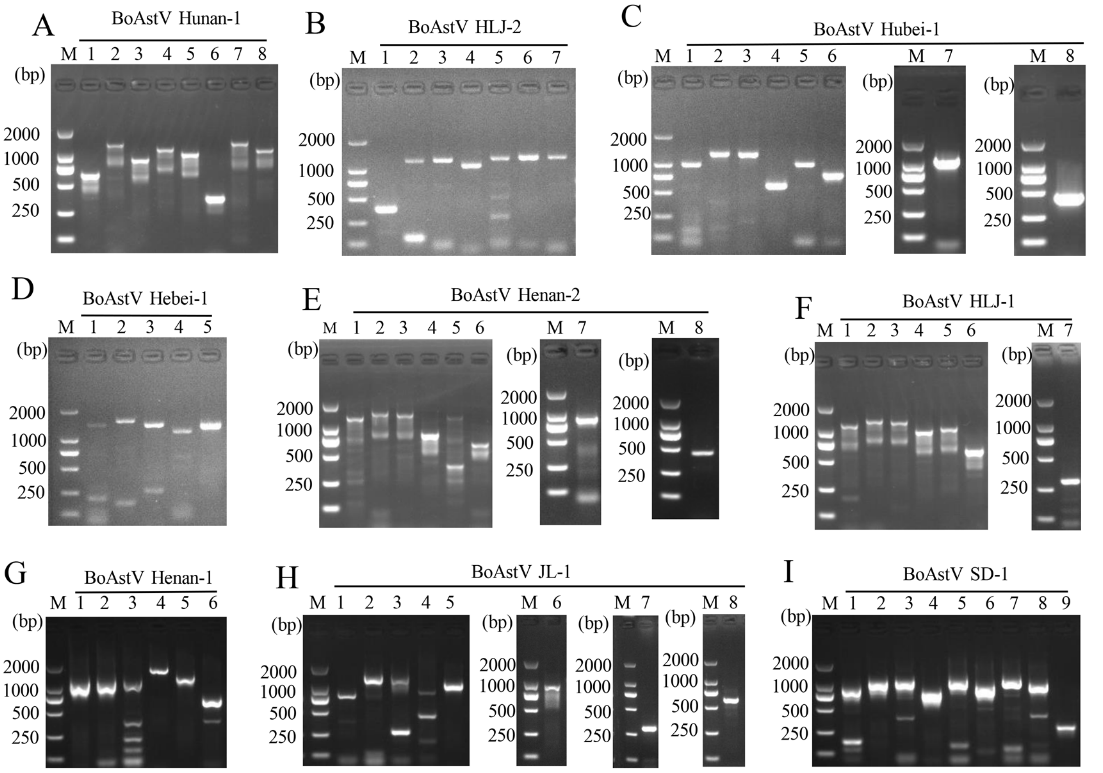

Figure S1: The complete genomes of nine BoAstV strains were verified by RT-PCR. The panels A, B, C, D, E, F, G, F and I were the verification results of BoAstV Hunan-1, BoAstV HLJ-2, BoAstV Hubei-1, BoAstV Hebei-1, BoAstV Henan-2, BoAstV HLJ-1, BoAstV Henan-1, BoAstV JL-1 and BoAstV SD-1, respectively. The nearly complete genome of each strain was divided into 5-9 fragments for RT-PCR. In the verification result of each strain, the number in each lane corresponds to the fragment number (Table S1). The RT-PCR products were analyzed by electrophoresis on 2% agarose gels. Product sizes amplified by various primer sets are shown in Table S1. Lane M represents DL2000 DNA ladder.

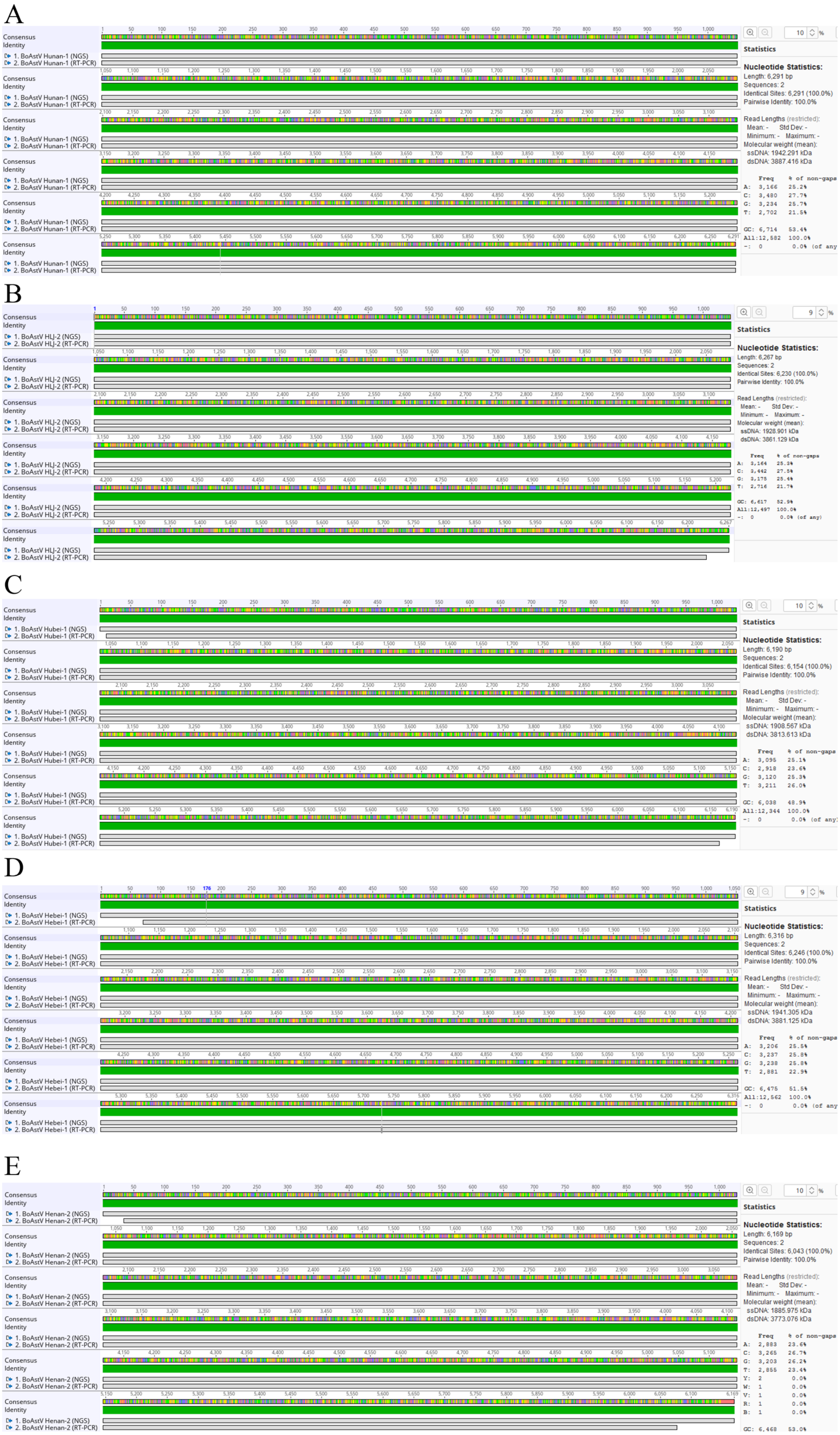
A
Consensus
Identity
1. BoAstV Hunan-1 (NGS)
2. BoAstV Hunan-1 (RT-PCR)
Statistics
Nucleotide Statistics:
Length: 6,291 bp
Sequences: 2
Identical Sites: 6,291 (100.0%)
Pairwise Identity: 100.0%
ssDNA: 1942.291 kDa
dsDNA: 3887.416 kDa
B
1. BoAstV HLJ-2 (NGS)
2. BoAstV HLJ-2 (RT-PCR)
Length: 6,267 bp
Sequences: 2
Identical Sites: 6,230 (100.0%)
Pairwise Identity: 100.0%
ssDNA: 1928.901 kDa
dsDNA: 3861.129 kDa
C
1. BoAstV Hubei-1 (NGS)
2. BoAstV Hubei-1 (RT-PCR)
Length: 6,190 bp
Sequences: 2
Identical Sites: 6,154 (100.0%)
Pairwise Identity: 100.0%
ssDNA: 1908.567 kDa
dsDNA: 3813.613 kDa
D
1. BoAstV Hebei-1 (NGS)
2. BoAstV Hebei-1 (RT-PCR)
Length: 6,316 bp
Sequences: 2
Identical Sites: 6,246 (100.0%)
Pairwise Identity: 100.0%
ssDNA: 1941.305 kDa
dsDNA: 3881.125 kDa
E
1. BoAstV Henan-2 (NGS)
2. BoAstV Henan-2 (RT-PCR)
Length: 6,169 bp
Sequences: 2
Identical Sites: 6,043 (100.0%)
Pairwise Identity: 100.0%
ssDNA: 1885.975 kDa
dsDNA: 3773.076 kDa

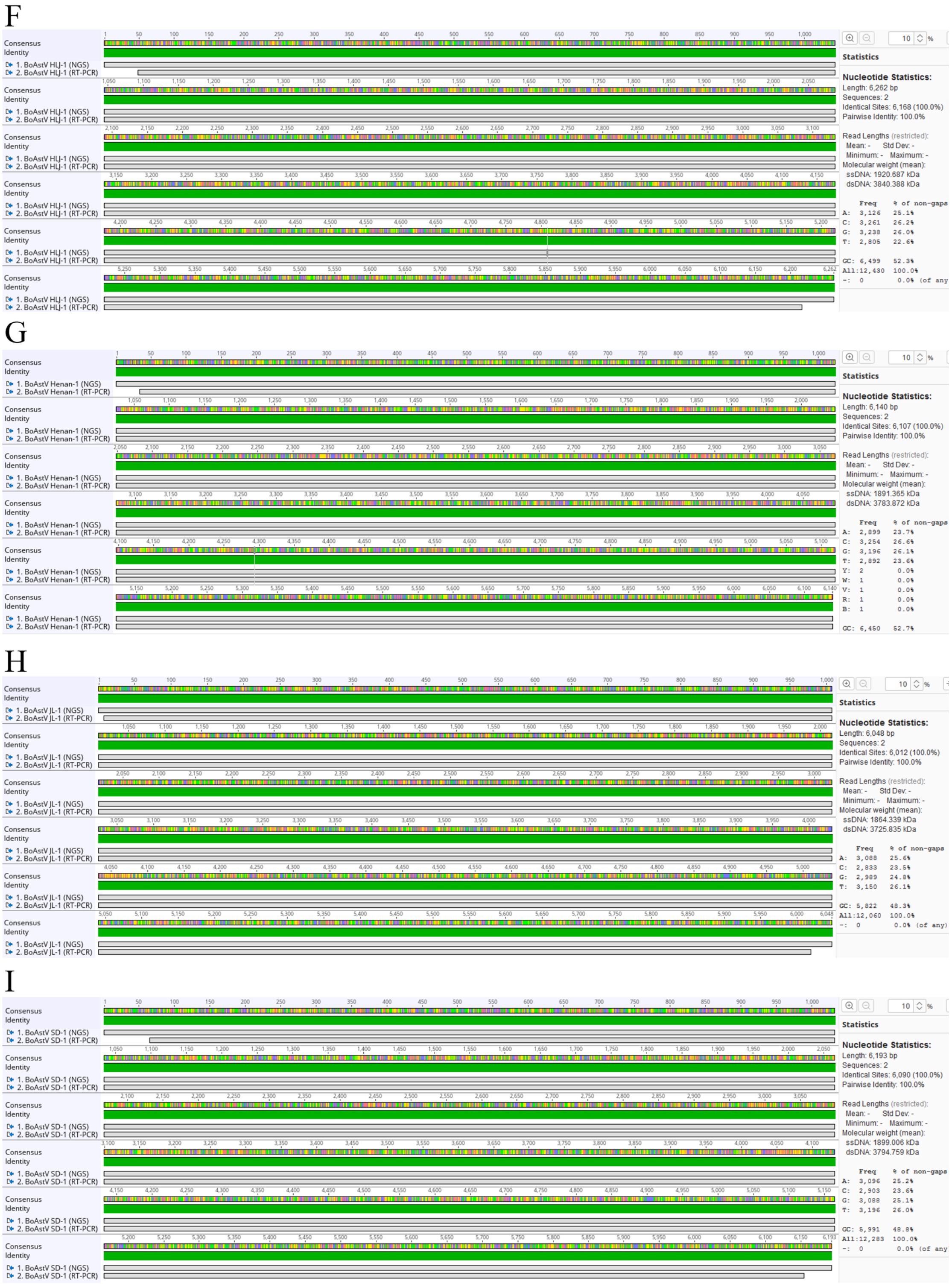

Figure S2: The identity of the genomes obtained by the next-generation sequencing (NGS) and RT-PCR/product sequencing. Figures A, B, C, D, E, F, G, F and I were the alignment and identity results of BoAstV Hunan-1, BoAstV HLJ-2, BoAstV Hubei-1, BoAstV Hebei-1, BoAstV Henan-2, BoAstV HLJ-1, BoAstV Henan-1, BoAstV JL-1 and BoAstV SD-1, respectively.

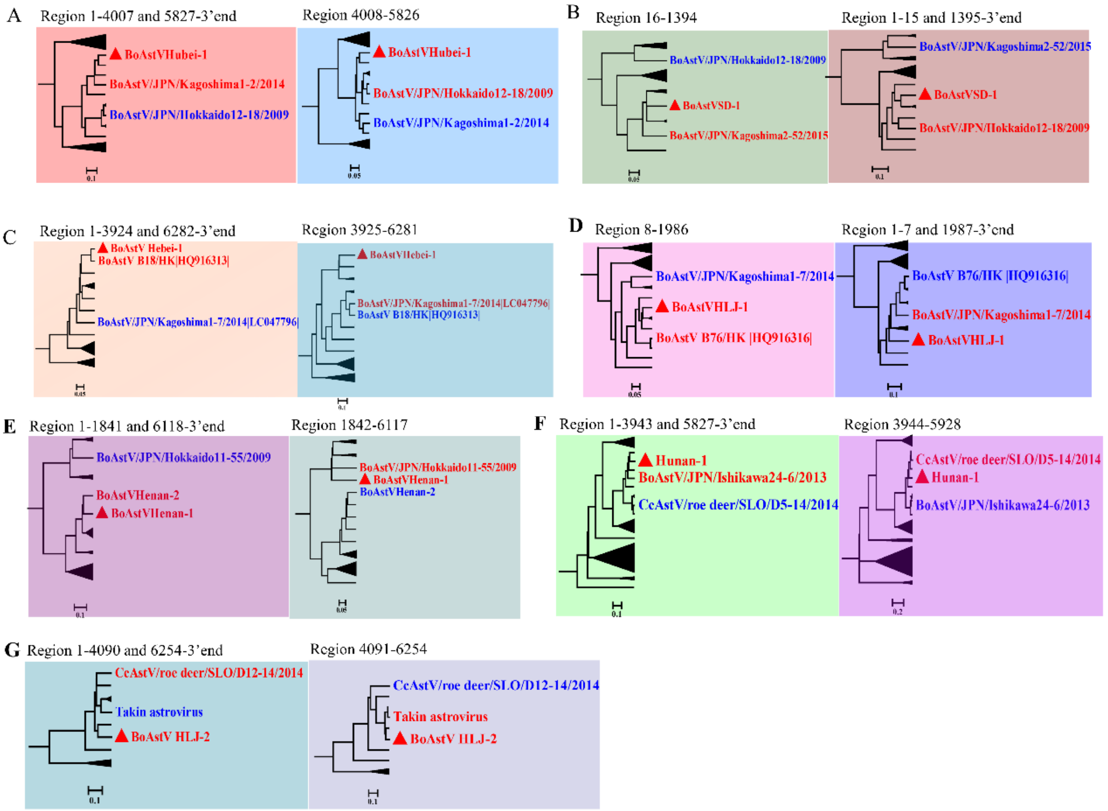

Figure S3: Recombination events were further confirmed by phylogenetic analyses of the different parental regions. Phylogenies of parental regions are in the same color as each genome was shown in A (BoAstV Hubei-1), B (BoAstV SD-1), C (BoAstV Hebei-1), D (BoAstV HLJ-1), E (BoAstV Henan-1), F (BoAstV Hunan-1) and G (BoAstV HLJ-2) respectively.
